# Targeting the tumor vasculature with engineered cystine-knot miniproteins

**DOI:** 10.1038/s41467-019-13948-y

**Published:** 2020-01-15

**Authors:** Bonny Gaby Lui, Nadja Salomon, Joycelyn Wüstehube-Lausch, Matin Daneschdar, Hans-Ulrich Schmoldt, Özlem Türeci, Ugur Sahin

**Affiliations:** BioNTech SE, Mainz, Germany

**Keywords:** Chemical modification, Peptides, Fluorescence imaging, Cancer imaging, High-throughput screening

## Abstract

The extra domain B splice variant (EDB) of human fibronectin selectively expressed in the tumor vasculature is an attractive target for cancer imaging and therapy. Here, we describe the generation and characterization of EDB-specific optical imaging probes. By screening combinatorial cystine-knot miniprotein libraries with phage display technology we discover exquisitely EDB-specific ligands that share a distinctive motif. Probes with a binding constant in the picomolar range are generated by chemical oligomerization of selected ligands and fluorophore conjugation. We show by fluorescence imaging that the probes stain EDB in tissue sections derived from human U-87 MG glioblastoma xenografts in mice. Moreover, we demonstrate selective accumulation and retention of intravenously administered probes in the tumor tissue of mice with U-87 MG glioblastoma xenografts by in vivo and ex vivo fluorescence imaging. These data warrants further pursuit of the selected cystine-knot miniproteins for in vivo imaging applications.

## Abstract (web summary)

Cystine-knot miniprotein are small, highly stable, disulfide-rich peptides with increasing potential as drugs and tumor imaging agents. Here the authors develop cystine-knot miniproteins targeting the vascular tumor marker EDB, and use them as probes for in vivo tumor vasculature imaging.

## Introduction

Computed tomography and magnetic resonance imaging are routinely used in cancer diagnosis and for monitoring of treatment outcome^1^. Targeted molecular imaging is emerging as an attractive complementary technology, as it is capable of visualizing tumors with high precision, specificity, and resolution by using molecular reagents directed against cancer-associated proteins^2^. Cell surface molecules associated with tumor angiogenesis that are expressed at high levels in the tumor blood vessels and hence are easily accessible from the bloodstream are considered as attractive targets^3^.

Fibronectin is a broadly expressed component of the extracellular matrix. Its splicing isoform, containing the extra domain B (EDB), is overexpressed during embryonic development and in a variety of solid tumors including brain cancer, whereas it is virtually undetectable in normal adult tissues^4,5^. EDB is a type III domain of 91 amino acids inserted between fibronectin (FN) domains 7 and 8 of the oncofetal fibronectin isoform^6,7^. The EDB sequence is highly conserved across species and the orthologous protein sequences in mice, rats, rabbits, and humans are completely identical^5^.

Being a selective tumor marker, EDB has been explored as a target for cancer imaging and therapy in early clinical trials^8^. L19, the most advanced EDB-selective ligand thus far, was isolated from an antibody phage display library by Neri and colleagues^9^. The L19 antibody has been investigated for various diagnostic and therapeutic applications^10–13^. As the large molecular size and complex folding of antibodies pose manufacturing challenges^14^, development of smaller EDB-specific ligands is being explored. These include ligands with nanomolar-binding affinities based on Fynomers, Anticalins, and an ubiquitin-derived protein selected from combinatorial phage libraries^15,16,17^. Moreover, two EDB-recognizing peptides have been generated and conjugated with fluorescent dyes for preclinical in vivo imaging studies. One study reports effective visualization of human prostate tumor xenografts with a cyclic nonapeptide called ZD2^18^. In another study, artificial high-affinity peptides (so-called aptides) have been shown to specifically target human glioblastoma tumor xenografts^19^. Altogether, these data have further boosted the interest in the concept of EDB targeting probes^8^.

In recent years, the cystine-knot miniprotein or knottin family with its superior pharmacological properties has gained particular attention for in vivo imaging and targeted drug delivery^20–22^. Cystine-knot miniproteins are small (30–50 amino acids in size) disulfide-rich peptides with extraordinarily high thermal, proteolytic, and chemical stability. Their simple architecture allows convenient chemical manufacturing and site-directed conjugation of, e.g. fluorophores, chelators, or toxins^23,24^. The exposed surface loops of cystine-knot miniproteins tolerate sequence modifications and insertions, facilitating the generation of target-binding capabilities via loop grafting or directed evolution based protein design. Cystine-knot miniprotein family members, including *Momordica cochinchinensis* trypsin inhibitor II (MCoTI-II), *Spinacia oleracea* trypsin inhibitor (SOTI), and *Ecballium elaterium* trypsin inhibitor II (EETI), have been engineered as specific binders against a variety of target proteins^25^.

This study describes the characterization of EDB-binding cystine-knot miniproteins, which are discovered by screening of a combinatorial phage display library based on an open chain variant of the trypsin inhibitor II from *Momordica cochinchinensis* (oMCoTI-II). MC-FN-010 and its derivative MC-FN-016 are selected for oligomerization and fluorescent dye conjugation to obtain trimeric imaging probes. These probes show specific in vivo tumor targeting properties in a glioblastoma xenograft mouse model, while they have low overall background signals. Our findings demonstrate the high potential of cystine-knot miniproteins for development of molecular imaging agents.

## Results

### Discovery of EDB-specific cystine-knot miniproteins

**Fig. 1:**
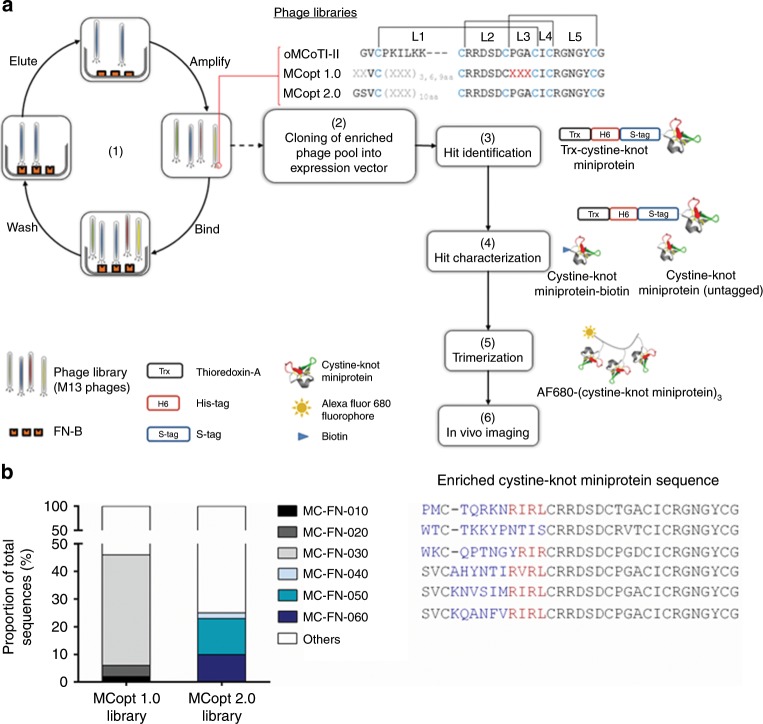
Enrichment of clones with a common sequence motif by library screening against EDB. **a** EDB-specific ligand selection and development of an imaging agent. (1) Three successive rounds of screening of MCopt 1.0 and MCopt 2.0 phage libraries (both based on the oMCoTI-II sequence framework) were performed against a hexahistidine (H6)-tagged single EDB-domain (FN-B) fragment. Disulfide bonds (brackets) between cysteine residues (blue), randomized positions for any random amino acid except cysteine (X in gray), and amino acid substitutions to 50% (X in red) are indicated. L1 to L5 represent the loop positions. (2) Cystine-knot miniprotein sequences were cloned into expression vector for Trx-cystine-knot miniprotein production. (3) Hit identification of individual clones was performed by ELISA-based binding analysis (Trx-cystine-knot miniprotein), determination of expression rate, and sequencing. (4) Hits were characterized with regard to affinity (with untagged cystine-knot miniprotein), specificity (Trx-cystine-knot miniprotein, cystine-knot miniprotein-biotin), and functionality (Trx-cystine-knot miniprotein). (5) Trimerization of lead cystine-knot miniprotein candidates and Alexa Fluor 680 fluorophore conjugation was performed to allow (6) imaging of tumor vasculature in vivo in a mouse model xenografted with a human glioblastoma cell line. **b** Enrichment of cystine-knot miniprotein sequences after three screening rounds of phage display libraries MCopt 1.0 and MCopt 2.0. Variable amino acids (blue letters) and the common R-I/V-R-(L) motif (red) are indicated.

For the selection of EDB-specific cystine-knot miniproteins, two different M13 phage libraries based on the open chain sequence of oMCoTI-II^26^ were used. The MCopt 1.0 library comprises sequences with randomized amino acids in the first loop, scattered positions in the third loop, and two variable residues upstream of the first cysteine, and is presented via the pVIII major coat protein, resulting in a polyvalent type of display. The MCopt 2.0 library, in contrast, is displayed via the minor coat protein (pIII) and contains a randomized stretch of 10 amino acids in the first loop only (Fig. 1a).

For the screening and the hit identification process we used a protein fragment representing the single EDB domain (FN-B). Hexahistidine (H6)-tagged FN-B was recombinantly expressed in *E. coli* and purified via immobilized metal ion affinity chromatography (IMAC) and size exclusion chromatography (SEC) to a purity of >90% (Supplementary Fig. 1a). In addition, EDB flanked by its surrounding type III domains (FN-67B89) and an analogous variant without inserted EDB (FN-6789), mimicking the respective epitope in healthy tissues, were generated as control proteins for downstream assays. Identity was confirmed by detecting the C-terminal H6-tag (Supplementary Fig. 1b). Native folding of FN-67B89 was verified in a enzyme-linked immunosorbent assay (ELISA)-based assay with a monoclonal antibody (BC-1), which distinguishes between fibronectin containing EDB and fibronectin without EDB^27^ (Supplementary Fig. 1b).

Both phage libraries were screened in three consecutive rounds against biotinylated FN-B and 46 single clones were selected for sequencing. Screening of the MCopt 1.0 library resulted in strong enrichment of one single cystine-knot miniprotein (MC-FN-030) comprising 40% of all sequences in the pool. Two other frequent clones were identified, representing 4% and 2% (MC-FN-020, MC-FN-010), respectively, of the repertoire (Fig. 1b). The sequences harvested from the third screening round were fused N-terminally to Thioredoxin-A and to a H6- and S-tag by subcloning and the respective Trx-cystine-knot miniproteins were expressed in a 96-well mini scale format (Fig. 1a). Binding of proteins in this pool to FN-B was tested in an ELISA-based assay and the expression rate of each clone was determined via 96-well SDS-PAGE (E-PAGE™) analysis. Based on the derived signal-to-noise ratio and the expression value, we calculated a ranking score for each Trx-cystine-knot miniprotein as a measure for FN-B interaction. Three candidates showed higher interaction with FN-B than with bovine serum albumin (BSA) as control. These sequences with confirmed FN-B binding were found to correspond to the enriched clones (Supplementary Table 1).

Screening of the second library, MCopt 2.0, resulted in enrichment of three different cystine-knot miniproteins to 13%, 10%, and 2% (MC-FN-050, MC-FN-060, MC-FN-040), respectively, of all sequences. Sequence analysis of all six clones derived from the two library screens revealed that five of these sequences (all except MC-FN-020) shared a common R-I/V-R-(L) motif at the C-terminal end of loop 1 (Fig. 1b). All six clones (those derived from MCopt 2.0 without further confirmation via binding ELISA) were subjected to further analysis.

**Fig. 2:**
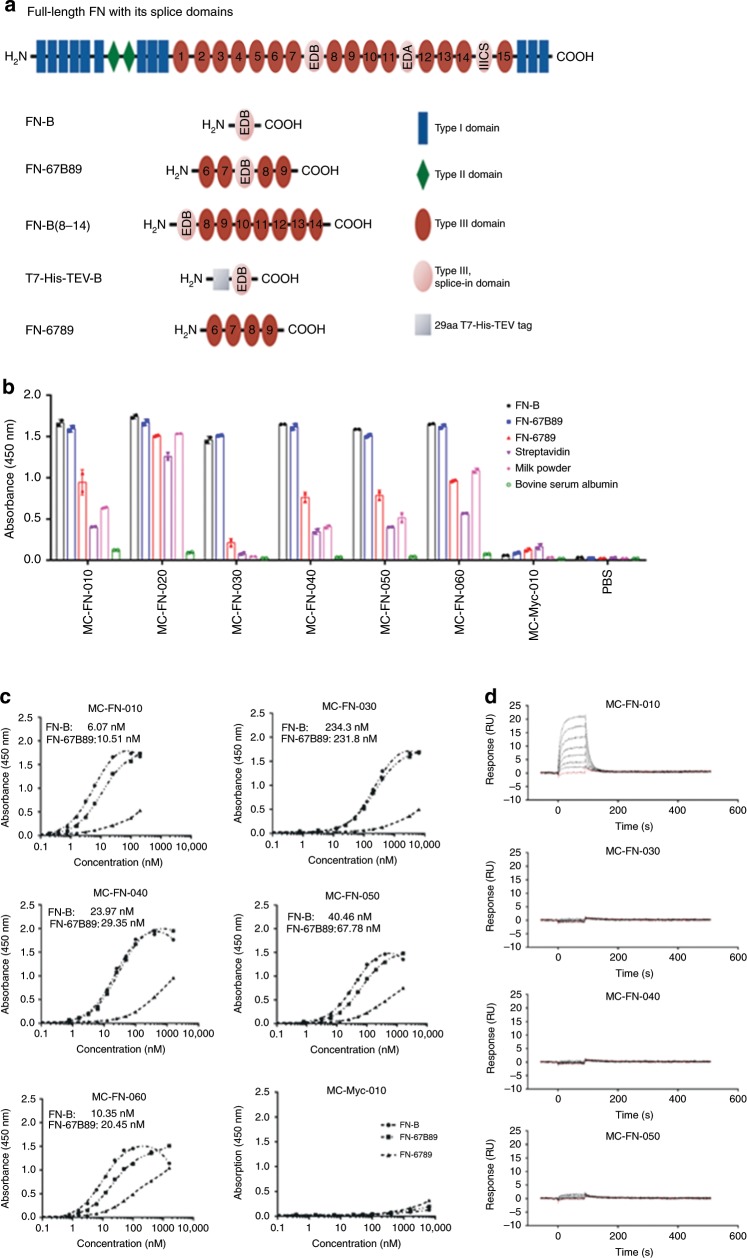
Discovery of cystine-knot miniproteins with specific binding to EDB. **a** EDB-containing protein fragments used in this study. The EDB domain distinguishes the oncofetal variant of fibronectin from the ubiquitously expressed fibronectin. FN-B (single domain), FN-67B89 (EDB flanked by the naturally surrounding type III domains), FN-B(8–14) (EDB protein with its C-terminal domains 8–13 and a part of domain 14), T7-His-TEV-B (N-terminally tagged EDB domain), and FN-6789 (the type III domains 6–9 without EDB). **b** ELISA-based specificity analysis of enriched cystine-knot miniproteins. In all, 200 nM of each Trx-cystine-knot miniproteins were applied to immobilized FN-B and FN-67B89 target proteins and to control proteins FN-6789, streptavidin, milk powder, and BSA (coated at 1 µg/well). Data are shown as mean ± SD. **c** EDB-binding curves of Trx-cystine-knot miniproteins were generated against FN-B and FN-67B89 targets and against FN-6789 control protein (coated at 1 µg/well). Binding was detected with an HRP-conjugated anti-S-tag antibody. ELISA was performed in duplicates in single measurement and with MC-Myc-010, harboring a Myc epitope in loop 1, serving as a negative control. The apparent KD-value was calculated based on non-linear regression fitting curves. **d** SPR sensorgrams of a multi-cycle kinetic binding analysis of selected cystine-knot miniproteins. Biotinylated FN-67B89 was captured on a streptavidin sensor chip and untagged cystine-knot miniproteins were analyzed with concentrations ranging from 50 to 1000 nM (curves from bottom down to top). The curves were referenced to a blank streptavidin control flow cell. Source data are provided as a Source Data file.

Binding specificity of the six candidates obtained by screening of the two libraries was analyzed by testing against various protein fragments containing the EDB domain alone or inserted in its natural or an artificial protein sequence, e.g. FN-B (with which the screening had been performed), FN-67B89, T7-TEV-B (a T7-His-TEV tag N-terminally flanked EDB domain), and FN-B(8–14) (EDB protein with its C-terminal domains 8–13 and a part of domain 14) (Fig. 2a) (for quality controls see Supplementary Fig. 1a). FN-6789 was used as off-target control, as fibronectin lacking EDB is expressed by many different cell types^28^, whereas the EDB variant is selective for tumor vasculature.

All clones were found to bind strongly to all recombinant fragments containing EDB (Fig. 2b, Supplementary Fig. 2). The five variants containing the common R-I/V-R-(L) motif showed low or moderate binding to off-target and control proteins, with MC-FN-060 having the highest background signal. For MC-FN-020, which does not contain the R-I/V-R-(L) motif, there was strong binding to the off-target control FN-6789 and the other control proteins (streptavidin and milk powder) except BSA. These data strongly indicate that the identified amino acid motif is relevant for selectivity of EDB binding.

Dose titration of the five R-I/V-R-(L)-motif-containing candidates revealed apparent KD values for binding to FN-B and FN-67B89, respectively, in the nanomolar range (6–235 nM) (Fig. 2c), with MC-FN-010 being the strongest binder. Binding to control FN-6789 was measurable at concentrations higher than one order of magnitude. Again, clone MC-FN-060 showed the highest non-specific binding to the FN-6789 off-target control.

These experiments had been performed with the tagged versions (Thioredoxin-A-, H6-, and S-tag) of the respective cystine-knot miniproteins. As for the purpose of being used as in vivo imaging tool any tags have to be removed, we next studied the binding ability of tag-free variants of the four strongest binders by surface plasmon resonance (SPR) analysis. Surprisingly, only MC-FN-010 showed detectable binding activity towards the FN-67B89 target protein at concentrations ranging from 50 to 1000 nM (Fig. 2d). Therefore, we focused on MC-FN-010 for further studies.

### Systematic analysis of the R-I/V-R-(L)-binding motif

**Fig. 3:**
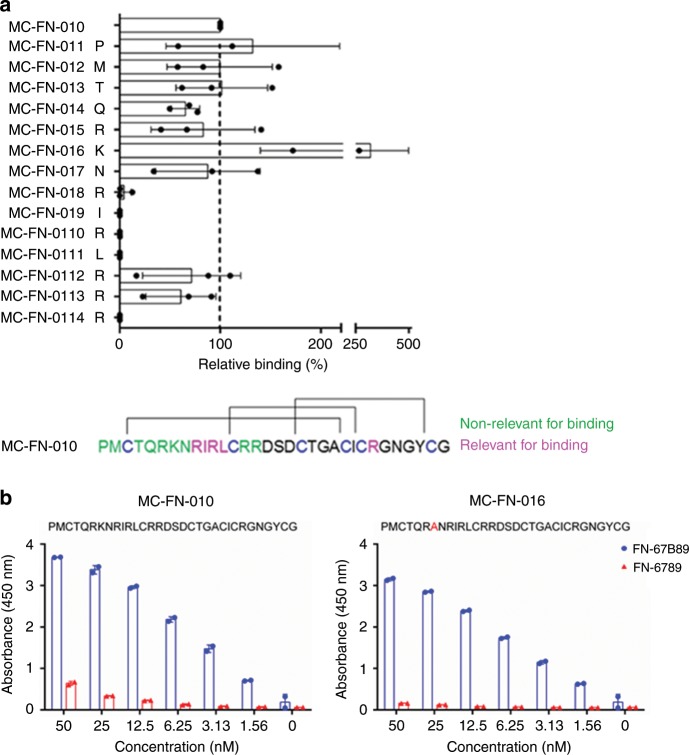
Dependency of EDB binding on the R-I/V-R-(L) motif and further sequence optimization. **a** Identification of functionally relevant amino acids in the MC-FN-010 sequence by alanine scanning mutagenesis. Trx-cystine-knot miniproteins (parental MC-FN-010 and alanine scanning variants) were incubated with pre-coated human FN-B. Binding was detected with HRP-conjugated anti-S-tag antibody. Relative binding of each variant was calculated by comparing the respective apparent binding constant to parental MC-FN-010. Error bars represent mean ± SD of three independent duplicate measurements. EDB binding relevant amino acid residues of parental MC-FN-010. Disulfide bonds (brackets) between cysteine residues (blue), residues relevant (magenta), or at best marginally relevant (green) for target binding are indicated. **b** ELISA-based binding analysis of MC-FN-010 and MC-FN-016 to human FN-67B89 and FN-6789 (1 µg/well). Trx-cystine-knot miniproteins were applied at different concentrations in duplicates and binding was detected with HRP-conjugated anti-S-tag antibody. Data shown as mean ± SD. Source data are provided as a Source Data file.

The occurrence of the amino acid motif R-I/V-R-(L) in the enriched and specifically binding cystine-knot miniproteins suggested involvement of this sequence in EDB binding. We performed a systematic alanine scanning mutagenesis of MC-FN-010, in which every single amino acid in the variable region and the arginine residues in loops 2 and 5 (L2 and L5, see Fig. 1a) were individually exchanged for alanine. All constructs were tested for binding to single domain FN-B (Fig. 3a). Four constructs with exchanges of single amino acids within the common motif positions had lower binding as compared to that of the respective original sequence. An additional alanine substitution in the fifth loop also led to reduced target interaction. In contrast, target binding was preserved in the seven constructs with alanine exchanges in the beginning of the sequence, indicating that those positions were not crucial for EDB binding. Together, these results confirm that the four amino acid residues in the first loop (RIRL), and the arginine residue in the fifth loop impact binding to EDB either directly or indirectly by affecting miniprotein conformation.

This analysis also identified mutagenized variants, which were comparable with regard to binding to the EDB domain. We leveraged these data for selection of a second development candidate, with which we could also address that the lysine residue in loop 1 of MC-FN-010 may interfere with the selective linkage to primary amines required for full exploitation as an imaging reagent. MC-FN-016 had a primary amine only at the N-terminus, and was found to be comparable to MC-FN-010 with regard to its specificity for binding to FN-67B89 (Fig. 3b). The affinities of tag-free MC-FN-010 and MC-FN-016 towards biotinylated FN-67B89 as tested by SPR analysis were in the single-digit micromolar range with fast off-rates (Table 1, Supplementary Fig. 3a).

**Table 1:** SPR-binding analysis of cystine-knot miniprotein variants to EDB.

Cystine-knot miniprotein	Concentration (nM)	Kon (1/Ms)	Koff (1/s)	KD (nM)
MC-FN-010	50–4000	4.95 × 10^4^	0.07831	1580
MC-FN-016	62.5–4000	1.33 × 10^5^	0.1873	1410
AF680-(MC-FN-010)_3_	1.25–10	8.04 × 10^6^	3.47 × 10^−3^	0.431
AF680-(MC-FN-016)_3_	1.25–10	2.56 × 10^6^	2.31 × 10^−3^	0.902
(MC-FN-010)_3_	1.25–10	4.82 × 10^4^	1.84 × 10^−4^	3.82
AF680-(MC-FN-0115)_3_	1.25–10	0	0	0

Kinetic parameters of EDB-specific cystine-knot miniprotein variants resulting from surface plasmon resonance analysis. Biotinylated human FN-67B89 was immobilized to a streptavidin sensor to determine affinity of MC-FN-010, MC-FN-016, AF680-(MC-FN-010)_3_, AF680-(MC-FN-016)_3_, and (MC-FN-010)_3_ using two-fold serial dilutions. Kinetic parameters were calculated using a 1:1 Langmuir fitting model applied to generated sensorgrams (Supplementary Fig. 3)

### Tumor vasculature targeting with cystine-knot miniproteins

Glioblastomas are known to express the fibronectin EDB isoform in their vasculature^29^ and cell line models derived from this tumor type are commonly used for in vitro and in vivo characterization of EDB-binding ligands^30,31^. We resorted to a mouse model based on inoculating the human glioblastoma cell line U-87 MG subcutaneously into the flanks of Fox n1/nu mice to obtain xenograft tumors for ex vivo analysis and in vivo studies.

**Fig. 4:**
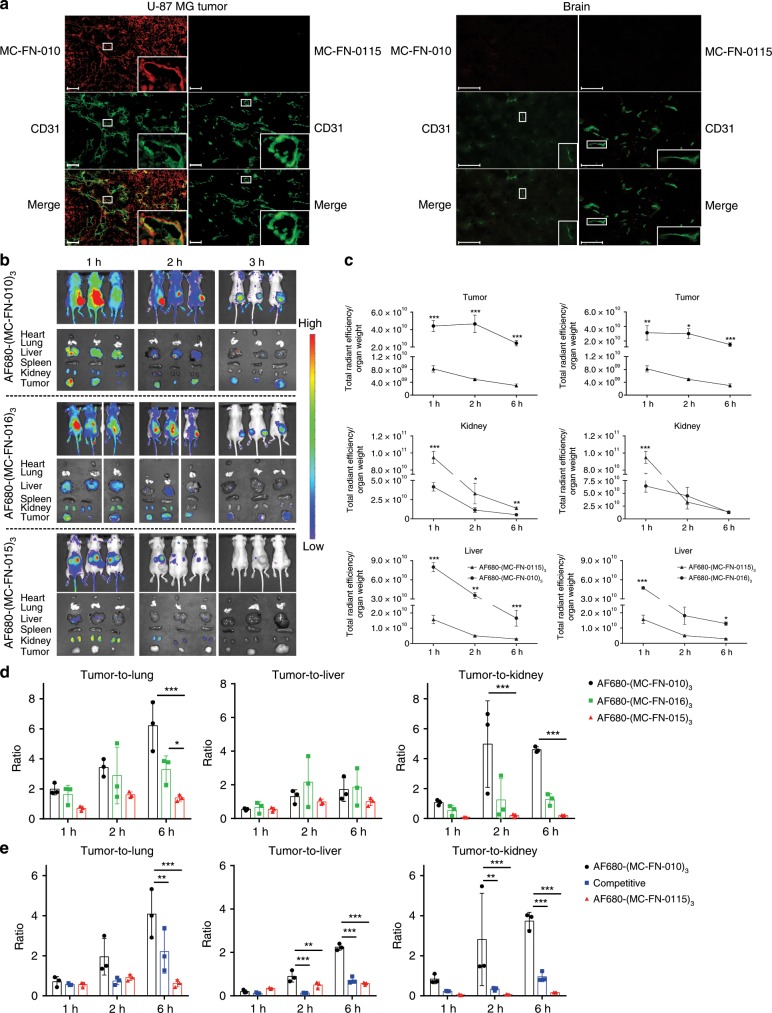
Targeting the tumor vasculature with selected cystine-knot miniproteins. **a** Specific binding of MC-FN-010 to tissue sections derived from the human U-87 MG glioblastoma cell line grown as mouse xenograft tumor. Representative immunofluorescence staining of U-87 MG tumor tissues and normal mouse brain with EDB ligand MC-FN-010 and negative control MC-FN-0115. Tissue sections (6 µm) were stained with tetramerized cystine-knot miniprotein-biotin/strepatividin-Cy3 complex (red) and an anti-CD31 antibody to visualize vasculature (green). Scale bars, 100 µm. **b** In vivo and ex vivo imaging of U-87 MG bearing mice. Tumors derived from human U-87 MG cells injected s.c. in flanks of mice were imaged after i.v. application of 3.34 nmol AF680-(MC-FN-010)_3_, AF680-(MC-FN-016)_3_ (EDB binder), and control AF680-(MC-FN-0115)_3_. Mice (*n* = 3) were stratified according to their tumor sizes. **c** Kinetics of fluorescence signals in different organs quantified and normalized to the respective organ/tumor weight. Each of the two cystine-knot miniproteins was compared to the control (AF680-(MC-FN-010)_3_ vs. AF680-(MC-FN-0115)_3_ and AF680-(MC-FN-016)_3_ vs. AF680-(MC-FN-0115)_3_, respectively) in a given organ at a given time point. **d** Kinetics of tumor-to-lung, tumor-to-liver, and tumor-to-kidney ratio. Ratios resulting from ex vivo imaging were derived from normalized fluorescence signals after i.v. injection of 3.34 nmol AF680-(MC-FN-010)_3_, AF680-(MC-FN-016)_3_, and AF680-(MC-FN-0115)_3_ at 1, 2, and 6 h. **e** Specific tumor targeting of AF680-(MC-FN-010)_3_ in a competitive experiment. Tumor-to-lung, tumor-to-liver, and tumor-to-kidney ratio of ex vivo imaging are calculated on the basis of the normalized fluorescence signals to the respective tumor weight at 1, 2, and 6 h (Supplementary Fig. 6). All data are shown as mean ± SD of triplicates. Statistical significance was calculated with two-way ANOVA (*p < 0.05; **p < 0.01; ***p < 0.001). Source data are provided as a Source Data file.

First, we tested MC-FN-010 staining on tissue sections derived from these xenograft tumors. MC-FN-0115 was used as non-binding negative control (alanine substitutions in three positions; PMCTQRANRIAACRRDSDCTGACICRGNGYCG) (Supplementary Fig. 4) and biotinylated MC-FN-010 and MC-FN-0115 were tetramerized with Cy3-labeled streptavidin to increase avidity. On U-87 MG tumor sections tetramerized MC-FN-010-bio bound almost selectively to areas around tumor vessels and co-localized with the vascular marker CD31, a surface protein ubiquitously expressed on endothelial cells, whereas MC-FN-0115-bio did not stain these sections at all. In addition, tetramerized MC-FN-010-bio was found to localize in perivascular areas. Normal mouse brain sections were not stained by MC-FN-010-bio (Fig. 4a), indicating specificity for tumor vasculature.

Next, for assessing MC-FN-010 and MC-FN-016 in vivo, the cystine-knot miniproteins were chemically trimerized by oxime ligation and tagged with a near-infrared fluorescent dye, Alexa Fluor 680 (Fig. 1a). Oligomerization profoundly improved affinity of both EDB-binding miniproteins. The trimers ((MC-FN-010)_3,_ AF680-(MC-FN-010)_3_, and AF680-(MC-FN-016)_3_) exhibited binding constants in the three-digit picomolar range and had remarkably slower off-rates as compared to the monomeric variants (Table 1, Supplementary Fig. 3b). EDB-binding properties of the cystine-knot miniproteins were not altered by trimerization as confirmed by immunofluorescence staining of tumor and normal brain tissue sections (Supplementary Fig. 5).

U-87 MG xenografted mice were injected intravenously with 3.34 nmol AF680-(MC-FN-010)_3_, AF680-(MC-FN-016)_3_, or the negative control AF680-(MC-FN-0115)_3_, and whole-body imaging and ex vivo measurement of resected organs was performed. In a parallel experiment, mice were injected with 10-fold excess of unlabeled (MC-FN-010)_3_ as competitor along with 3.34 nmol AF680-(MC-FN-010)_3_ to compare the kinetics of tumor fluorescence intensity to the in vivo experiment without competition. Both AF680-(MC-FN-010)_3_ and AF680-(MC-FN-016)_3_ but not the control variant strongly accumulated in the tumor within the first hours and remained significantly enriched over the recorded 6 h time period (Fig. 4b, c). As a consequence, analysis of resected tissues showed that tumor-to-organ ratios of AF680-(MC-FN-010)_3_ and AF680-(MC-FN-016)_3_ for organs, which are of relevance as frequent sites of metastasis in clinical imaging, increased over time and enabled improved capture of the specific signal (Fig. 4d, Table 2). As demonstrated by ex vivo imaging, the tumor signals in mice treated with the competitor were substantially reduced at each time point (Supplementary Fig. 6). The resulting tumor-to-organ ratios significantly increased over time demonstrating a strong EDB specificity of AF680-(MC-FN-010)_3_ (Fig. 4e).

**Table 2:** Biodistribution of cystine-knot miniproteins in human U-87 MG xenograft mouse tumors.

Cystine-knot miniprotein	Tumor-to-organ ratio	1 h	2 h	6 h
AF680-(MC-FN-010)_3_	Tumor-to-heart	8.57 ± 2.35	18.09 ± 10.32	14.77 ± 2.20
AF680-(MC-FN-010)_3_	Tumor-to-lung	1.98 ± 0.37	3.43 ± 0.58	6.21 ± 1.64
AF680-(MC-FN-010)_3_	Tumor-to-liver	0.55 ± 0.06	1.30 ± 0.40	1.71 ± 0.71
AF680-(MC-FN-010)_3_	Tumor-to-spleen	4.69 ± 1.33	9.53 ± 2.12	8.94 ± 1.46
AF680-(MC-FN-010)_3_	Tumor-to-kidney	1.06 ± 0.17	4.97 ± 2.89	4.60 ± 0.19
AF680-(MC-FN-016)_3_	Tumor-to-heart	12.86 ± 6.70	13.58 ± 10.53	9.88 ± 3.02
AF680-(MC-FN-016)_3_	Tumor-to-lung	1.62 ± 0.62	2.89 ± 1.89	3.29 ± 0.91
AF680-(MC-FN-016)_3_	Tumor-to-liver	0.66 ± 0.33	2.15 ± 1.51	1.86 ± 1.10
AF680-(MC-FN-016)_3_	Tumor-to-spleen	5.37 ± 2.67	7.44 ± 3.86	7.31 ± 1.98
AF680-(MC-FN-016)_3_	Tumor-to-kidney	0.54 ± 0.34	1.25 ± 1.41	1.27 ± 0.39
AF680-(MC-FN-0115)_3_	Tumor-to-heart	3.19 ± 0.51	4.39 ± 1.57	3.11 ± 0.69
AF680-(MC-FN-0115)_3_	Tumor-to-lung	0.70 ± 0.16	1.61 ± 0.19	1.40 ± 0.21
AF680-(MC-FN-0115)_3_	Tumor-to-liver	0.54 ± 0.13	0.99 ± 0.16	1.00 ± 0.23
AF680-(MC-FN-0115)_3_	Tumor-to-spleen	1.96 ± 0.32	2.57 ± 0.62	1.91 ± 0.41
AF680-(MC-FN-0115)_3_	Tumor-to-kidney	0.09 ± 0.02	0.20 ± 0.11	0.21 ± 0.03

Tumor-to-organ ratios of AF680-(MC-FN-010)_3_, AF680-(MC-FN-016)_3_, and AF680-(MC-FN-0115)_3_ were calculated based on the fluorescence signals (normalized to the weight) after i.v. injection of 3.34 nmol samples at 1, 2, and 6  h (*n* = 3). Data are shown as mean ± SD

We compared the in vivo tumor targeting of AF680-(MC-FN-010)_3_ to two previously described EDB-binding peptides (aptide^17^ and nonapeptide^19^). In the comparative study, 3.34 nmol AF680-(MC-FN-010)_3_, AF680-aptide, and AF680-nonapeptide were intravenously administered to U-87 MG bearing mice (Supplementary Fig. 7, Supplementary Table 2). In accordance to our previous observations, AF680-(MC-FN-010)_3_ was strongly enriched in the tumors and tumor-to-organ ratios increased markedly over time. In contrast, AF680-aptide was predominantly detected in the liver and kidney with low tumor-to-organ ratios. For detectability of fluorescence signals of AF680-nonapeptide in the tumor at 1 h post-injection, a higher dose (10 nmol) of the probe had to be administered (Supplementary Fig. 8).

In aggregate, these data warrants to further explore the identified EDB-specific cystine-knot miniproteins for use as imaging agents for tumor targeting.

## Discussion

Targeted tumor imaging with peptide- or protein-based molecular probes may improve cancer diagnosis, staging, and treatment monitoring^32^. The selective expression of the EDB-containing isoform of Fibronectin in tumor-associated neovasculature^7^ makes it an ideal target for ligand-based delivery of drug molecules. Derivatives of the EDB-binding human L19 antibody have been widely used as a molecular system to deliver radioisotopes or cytokines in cancer imaging and therapy^33–35^. Several L19-based approaches are currently under clinical investigation with promising early results^36,37^. This together with the fact that cystine-knot miniproteins entail a broad range of favorable characteristics as compared to a full antibody format encouraged us to generate EDB-binders based on this scaffold.

In this study we present the development of cystine-knot peptide-based ligands with high specificity for fibronectin EDB and its preclinical evaluation as an in vivo imaging probe in a glioblastoma mouse model. The screening of two combinatorial libraries against an EDB-containing protein fragment resulted in strong enrichment of six clones, five of which shared the distinctive R-I/V-R-(L) motif. We characterized the identified hits and selected MC-FN-010 based on the robust affinity and specificity of its binding to EDB for further development. Mapping of the MC-FN-010-binding site through alanine scanning mutagenesis revealed that the amino acid residues R-I-R-L in the first loop and an arginine residue in the fifth loop are required for EDB-binding activity of the peptide. One may speculate that the consensus sequence together with amino acids located in loop 2 (R-R-D-S-D) and loop 5 (R-G-N-G-Y) forms a patch of positively charged residues that mediate binding to EDB mainly via electrostatic interactions. This hypothesis is supported by the dominance of negative charges with only two positively charged residues being represented within the 91 amino acids of EDB^38^.

The measured binding affinities of the parental MC-FN-010 and its derivative MC-FN-016 to FN-67B89 were in the single-digit micromolar range. As a higher binding strength is required for efficient tumor uptake and retention in vivo, in particular for small peptides that are excreted rapidly via the kidneys^39,40^, we oligomerized these ligands and conjugated the resulting trimers with a fluorescent dye. This resulted in apparent affinities in the picomolar range (~1500- to 3600-fold stronger as compared to the monomeric versions), comparable with the reported binding constants for the single-chain Fv L19 fragment^9^.

The ability of the trimerized and labeled formats of MC-FN-010 and MC-FN-016 to bind to EDB-expressing tumors in vivo was investigated in the U-87 MG xenograft mouse model. Both molecules were selectively taken up and maintained within the tumor over the entire recorded period of 6 h in contrast to a triple alanine mutant that served as a negative control. Signals observed in liver and kidney decreased over time. The uptake and retention of oMCoTI-II-based peptides in the kidneys was reported in other studies and attributed to a high content of arginine residues^41^. Benchmarking with previously described peptide-based binders, aptide^17^ and nonapeptide^19^, supported the favorable performance of the presented cystine-knot derived probes.

Taken together, the strong tumor uptake of these trimeric constructs and their favorable biodistribution indicate the suitability for diagnostic imaging applications. These EDB ligands may provide a platform, which can support a diversity of in vivo cancer imaging and targeting approaches including conjugation of radioactive tracers, use for PET or SPECT nuclear imaging techniques, and targeting of bioactive payloads.

## Methods

### Target expression, purification, and biotinylation

Recombinant human fibronectin EDB, either as a single domain (FN-B, Uniprot ID P02751, isoform 7, amino acid E1265-T1355) or flanked by its surrounding type III domains (FN-67B89, amino acid G1080-E1455), served as target protein in this study while a protein comprising domains 6–9 without EDB (FN-6789) was used as a control. All variants were expressed in *E. coli* with a c-terminal hexahistidine (H6) tag and purified via IMAC and SEC. For this, codon-optimized DNA sequences were synthesized by Thermo Fisher Scientific, cloned into the pET-21a expression vector (Novagen), and introduced into *E. coli* BL21 (DE3) cells (Agilent). Proteins were produced at volumes of 750 mL at 30 °C, 120 rpm until an OD_600_ of approximately 0.7 was reached. For induction of production, 750 μL 1 M IPTG were added to the main culture and incubated at 25 °C, 120 rpm. overnight. Cells were harvested, re-suspended in 10 mL equilibration buffer (20 mM Tris-HCl pH 8.0, 10% glycerol, 500 mM NaCl, 10 mM imidazole), and lysed by sonification (Branson Digital Sonifier 250). The supernatant was purified by IMAC with a 1 mL HisTrap column (GE Healthcare) using an ÄKTAprime™ plus system (GE Healthcare) and a linear gradient from 10–500 mM imidazole in 20 min. Subsequently, target protein containing fractions were combined, dialyzed against phosphate-buffered saline (PBS) (14 mM NaCl, 2.7 mM KCl, 10 mM Na_2_HPO_4_, 1.8 mM KH_2_PO_4_, pH 7.5) at 4 °C overnight, and further purified by size exclusion chromatography using a HiLoad 26/600 Superdex 200 pg (for FN-67B89 and FN-6789) or 75 pg (for FN-B) column (GE Healthcare). Final purified proteins were analyzed via SDS-PAGE, analytical SEC, and via ELISA using the EDB-specific BC-1 antibody (ab154210, Abcam). Protein batch purity was furthermore determined via densitometric analysis using ImageQuant software from unmodified SDS-PAGE image. Proteins were stored in aliquots in PBS supplemented with 5% mannitol and 5% trehalose at −20 °C. FN-B, FN-67B89 and FN-6789 proteins were biotinylated via primary NH_2_-groups using EZ-Link Sulfo-NHS-LC-Biotin (Thermo Fisher Scientific) according to the manufacturer’s introductions.

### Quality control of FN-67B89 target protein

In all, 10 μg/mL target proteins were coated on a Maxisorp^TM^ 96-well plate in a volume of 100 μL via passive absorption under alkaline conditions, using 50 mM Na_2_CO_3_ pH 9.4 coating buffer. Coating of ELISA plates was performed overnight at 4 °C. After removal of coating buffer, the plates were washed three times with 300 μL PBS-T and 300 μL blocking buffer (3% BSA in PBS) was added. Blocking was performed for 2 h at RT. For primary incubation, plates were washed three times with 300 μL PBS-T and incubated for 1 h with 100 μL of BC-1 antibody diluted 1:1000 in PBS-T, at 4 °C. An HRP-conjugated anti-mouse antibody (554002, BD Pharmingen™) diluted 1:5000 in PBS was added after washing three times with PBS-T in 100 μL/well and incubated for 1 h at 4 °C. Plates were then washed three times with PBS-T and three times with PBS prior to detection of antigen–antibody complexes via HRP-mediated conversion of TMB substrate. For this, 100 μL TMB substrate was added to the wells and a blue color developed in proportion to the amount of analyte present in the sample. Color development was stopped by adding of 50 μL 0.2 M HCl per well and the specific absorbance was measured at 450 nm using an Infinite M200 PRO Microplate Reader (Tecan).

### Selection of EDB-specific ligands via phage display

Two different combinatorial libraries, which are based on oMCoTI-II were constructed using an M13 phagemid system. While in the MCopt 1.0 library loop 1 (including a length variation of 6, 9, and 12 amino acids) and loop 3 as well as the two amino acids at the N-terminus have been randomized, the MCopt 2.0 library was built via randomization of loop 1 with 10 amino acids only. Additionally, they are also different in the type of display as MCopt 1.0 is presented via the major coat protein pVIII and MCopt 2.0 via pIII. In total, three screening rounds based on streptavidin-coated (SA) magnetic beads were performed with each library. For each screening round 2 × 50 μL Dynabeads^®^ M-280 Streptavidin (Life Technologies) were transferred to a 2 mL tube each and washed with 1 mL TBS-T buffer (50 mM Tris, 150 mM NaCl, 0.1% Tween-20, pH 7.4). 100 µg  biotinylated FN-B in 200 μL TBS (50 mM Tris, 150 mM NaCl, pH 7.4) was added to the first tube, 200 μL TBS without target protein to the second tube (used for negative selection of phages) and beads were incubated on a rolling mixer for 20 min at 30 rpm. Tubes were placed back into the magnet, remaining solution was discarded, and the beads were washed twice with TBS-T. Beads were blocked with 2% milk powder (Carl Roth) in TBS for 1 h at 4 °C and 30 rpm. While biotinylated FN-B-coated SA beads were blocked for another 30 min, 7 × 10^13^ (first round) or 7 × 10^12^ (second and third round) phages were added to the uncoated SA beads in 1 mL 2% milk powder in TBS and incubated for 30 min at RT, 30 rpm (negative selection). The blocking solution of target-coated beads was discarded; beads were washed twice with TBS-T and phage supernatant from negative selection was added. Target-coated beads were incubated with the phage suspension for 1 h at RT and 30 rpm. Subsequently, unbound phages were washed off, washing the beads six times with TBS-T and twice with TBS. To elute bound phages, a pH-shift elution was performed, adding 50 μL 100 mM trimethylamine (TEA) to the washed beads. The TEA bead suspension was incubated for 6 min at max rpm, placed back into the magnet, and supernatant transferred to a fresh tube containing 100 μL 1 M Tris/HCl pH 7 for neutralization. A second elution step was performed by adding 50 μL 100 mM glycine (pH 2) to the target-coated beads and incubating the mixture for 10 min in a thermomixer at max rpm. Tubes were placed back into the magnet and the supernatant was transferred to the Tris/TEA mixture from the first elution. Phage eluate was used to infect exponentially growing *E. coli* XL1-Blue (Agilent) for phage pool amplification. In total, 1800 μL *E. coli* XL1-Blue cells at an OD_600_ of 0.5 were added to the eluate and incubated for 30 min at 37 °C without and for 30 min at 37 °C with agitation at 150 rpm. The infected XL1-Blue cells were then dispended on two large agar plates containing 100 µg/mL carbenicillin and 0.4% glucose and incubated overnight at 37 °C, before phage rescue was performed on the following day. For phage rescue, 4 mL LB medium was distributed per plate and cells scraped off with a cell scraper. A 50-mL culture was inoculated to a final OD_600_ of 0.2. Cells were grown until an OD_600_ of 0.5 and infected with 0.5 × 10^12^ VCSM13 helper phages (Agilent) for phage production from phagemid vector. *E. coli* XL1-Blue cells were incubated for 30 min, 37 °C, without agitation followed by 30 min, 37 °C, with agitation at 150 rpm. The bacterial suspension was centrifuged at 4500 × *g* for 10 min at RT and the supernatant was discarded. Bacterial pellets were taken up in 50 mL LB medium (supplemented with 100 µg/mL carbenicillin, 25 µg/mL kanamycin, 1 mM IPTG) for induction of protein production and *E. coli* phage production cultures grown overnight at 30 °C, 250 rpm. *E. coli* cells were harvested by centrifugation at 4500 × *g* for 15 min, 4 °C. 10 mL  PEG/NaCl solution (25% (w/v) polyethylene glycol, 15% (w/v) NaCl) were added to 40 mL of the phage containing supernatant, inverted, and placed on ice for 30 min. Phage particles were precipitated via centrifugation at 15,000 × *g*, 20 min at 4 °C. Supernatants were discarded and phage pellets were taken up in 1600 μL Tris/HCl (pH 8.0) for subsequent centrifugation at 15,000 × *g*, 10 min at 4 °C. The phage containing supernatant was added to 400 μL PEG/NaCl again and incubated on ice for 20 min. After the second PEG/NaCl precipitation, tubes were centrifuged at 15,000 × *g*, 15 min at 4 °C. Phage pellets were re-suspended in 800 μL Tris/HCl and heated to 65 °C for 15 min in a final purification step. Phage suspensions were finally centrifuged at RT, 15,000 × *g* for 10 min, supernatants were taken and phage particle concentration was determined photometrically using the dual wavelength modus OD_269_−OD_320_. Phage concentrations were calculated according to the nucleotide content and molar extinction coefficient of M13 phages^42^.

### Hit identification of selected cystine-knot miniproteins

In the identification process, enriched screening pool phagemid vectors derived from the third screening round were prepared in order to amplify cystine-knot miniprotein sequences by PCR. PCR-inserts were cloned into the expression vector pET-32-LibEx, a derivative of pET-32a (Novagen), to enable an expression of cystine-knot miniproteins as Thioredoxin-A fusion variants. This vector carries DNA sequences in successive order encoding for *E. coli* Thioredoxin-A to allow efficient disulfide bond formation in the cytoplasm in combination with an oxidoreductase knock-out strain^43^, an H6-tag for rapid purification, an S-tag for detection by an antibody, and a thrombin cleavage site to remove the fusion tag. DNA fragments encoding for cystine-knot miniprotein sequences were introduced downstream of the thrombin cleavage site into pET-32-LibEx vector via unique *Bam*HI and *Kpn*I restriction sites. Vectors were introduced into *E. coli* SHuffle^®^ T7 Express cells (New England BioLabs) via heat shock and the cells were plated on selective agar plates. Single colonies were picked, sequenced, and transferred to a 96-well plate for small-scale expression in 1 mL autoinduction medium (MagicMedia™; Thermo Fisher Scientific). Protein production was conducted overnight at 30 °C and 220 rpm. Cells were harvested by centrifugation at 3000 × *g* for 15 min, lysed via incubation in buffer (20 mM Tris, 2 mM MgCl_2_, 20 mM NaCl, pH 8) containing 0.1 mg/mL lysozyme (Merck Millipore) and 5 U/mL benzonase (Merck Millipore) combined with a freeze–thaw cycle, and heated for 10 min at 80 °C. After a final centrifugation (3000 × *g*, 15 min, 4 °C) to remove cell debris, the supernatant was collected for E-PAGE™ and binding analysis. E-PAGE^TM^ (Life Technologies) was used as a high throughput gel system to simultaneously analyze 96-probes in parallel for a quantification of produced and heat-purified proteins. E-PAGE^TM^ analysis was performed according to the manufacturer's instructions. ELISA was used as a technique to analyze protein–protein interactions. Cavities of 96-well microtiter plates (Nunc MaxiSorp™; Thermo Fisher Scientific) were coated with 1 µg FN-B, BSA (Eurobio), milk powder, streptavidin (Sigma Aldrich), T7-His-TEV-B (LD BioPharma), FN-B(8–14) (R&D Systems), lysozyme, ovalbumin (GE Healthcare Life Science), aldolase (GE Healthcare Life Science), or 0.6 µg anti-c-myc antibody (M4439; Sigma) overnight at 4 °C. The wells were washed three times with PBS-T (1× PBS with 0.1% (w/v) Tween-20), blocked with 1× Casein buffer (Sigma Aldrich) diluted in PBS for 2 h at RT, and washed again as described. 20 µL of supernatant containing the respective heat-purified fusion protein were added to 80 µL PBS-T or 200 nM of MC-Myc-010 fusion protein (wells with anti-c-myc antibody) diluted in PBS-T, applied to the cavities, and incubated for 1 h at 4 °C. After three times of washing with PBS-T, binding of the respective variant was detected with a horseradish peroxidase (HRP)-conjugated anti-S-tag antibody (ab18589 or ab19324; Abcam) 1:2000 diluted in PBS. Enzymatic reaction was measured with TMB as a chromogenic substrate and stopped with 0.2 M HCl after approximately 5 min. The measurement of absorbance at 450 nm was performed using an Infinite M200 PRO Microplate Reader (Tecan). In order to compare binding signals among different plates, ELISA signals were normalized to the internal plate control (c-myc antibody binding). Normalized FN-B signals were then referenced to normalized BSA signals to evaluate binding ability of selected cystine-knot miniproteins. In addition, the target-binding signals were correlated to the protein expression rate, resulting in a ranking value for the identification of hits.

### Recombinant cystine-knot miniprotein production

Recombinant protein production was carried out using *E. coli* SHuffle^®^ T7 Express cells carrying pET-32-LibEx vector encoding for the respective cystine-knot miniprotein sequence in a 750-mL scale at 30 °C, 120 rpm. After the culture reached an OD_600_ of approximately 0.7, induction of production was achieved by adding 750 μL 1 M IPTG and incubation at 25 °C, 120 rpm overnight. *E. coli* cells were harvested, re-suspended in 10 mL equilibration buffer, lysed by sonification, and heated to 80 °C for 10 min. After centrifugation of cell debris (15,000 × *g* for 30 min, 4 °C), the supernatant was purified by IMAC with a 1 mL HisTrap column using an ÄKTAprime™ plus system and a linear gradient from 10 to 500 mM imidazole in 20 min. The cystine-knot miniprotein fusion protein (Trx-cystine-knot miniprotein) containing fractions were collected and dialyzed against thrombin cleavage buffer (20 mM Tris, 150 mM NaCl, 1.5 mM CaCl_2_, and 5% (w/v) glycerol, pH 8.45) at 4 °C overnight. Trx-cystine-knot miniproteins were either directly used for ELISA-based assays or processed further in case that the untagged miniprotein was needed, e.g. for SPR analysis. For this, Trx-cystine-knot miniproteins were cleaved with 0.5 U of thrombin (Sigma Aldrich) per 1 mg protein and incubated at 37 °C overnight. Separation of cleaved protein fragments was performed by reverse phase chromatography with an Agilent 1260 Infinity Quaternary LC system (Agilent) and a 3 mL RESOURCE™ RPC column (GE Healthcare) using a linear gradient from 2% to 80% acetonitrile in H_2_O supplemented with 0.05% trifluoroacetic acid (TFA). Respective fractions containing cystine-knot miniprotein were lyophilized in a RVC 2-18-CD Plus rotational vacuum concentrator (Christ) as a final step. Amount of cystine-knot miniprotein was determined by weighing and the peptides were stored in lyophilized form at −20 °C. Identity was verified by mass spectrometry with a LCMS Single Quad G6130B System (Agilent Technologies) using a standard electrospray ionization protocol.

### Alanine scanning mutagenesis of selected MC-FN-010

For generation of alanine scanning MC-FN-010 derivatives mutations were either introduced via PCR or the whole coding sequence was assembled via direct synthesis of GeneArt™ Strings™ fragments (Thermo Fisher Scientific). Respective DNA fragments were cloned into pET-32-LibEx expression vector using unique *Bam*HI and *Kpn*I restriction sites and introduced into *E. coli* SHuffle^®^ T7 Express competent cells (New England BioLabs). All mutations were verified by DNA sequencing. The alanine scanning mutagenesis variants were expressed in 24-well format using 5 mL of selective autoinduction medium. Production and Trx-cystine-knot miniprotein purification were performed as described above for the 96-well format, but included a further purification step of the supernatant using HisPur™ Ni-NTA spin columns (Thermo Fisher Scientific). Binding ability and specificity of Trx-cystine-knot miniproteins to target and off-target protein was carried out with an antibody-based ELISA assay as described above.

### Surface plasmon resonance spectroscopy

Binding kinetics of monomeric and trimeric cystine-knot miniprotein ligands to its target protein was determined using a Biacore T-100 device (GE Healthcare Life Science) with PBS-T as running buffer. For this, the biotinylated FN-67B89 protein (200–300 µg/mL) was captured by binding to a flow cell of an SA sensor chip (GE Healthcare Life Science). To analyze monomeric ligands, an immobilized target density of maximum 750 response units (RU) was applied and for trimeric variants a RU of maximum 400 was aimed for. Binding analysis of monomeric ligands was performed using a multi-cycle kinetic method with concentrations ranging from 50 to 4000 nM. A cycle started with an association period of 90 s, followed by a dissociation period of 420 s and a final regeneration step. Kinetic measurement was conducted applying a flow rate of 20 µL/min. Trimeric variants were analyzed under the same association and dissociation conditions, but using the single-cycle kinetic measurement mode in a constant flow of 30 µL/mL. In this case the analyte concentration was between 1.25 and 10 nM. Binding kinetics and steady-state analysis were calculated using a global kinetic fit model (1:1 Langmuir, Biacore T-100 Evaluation Software; GE Healthcare Life Science).

### Immunofluorescence staining

Cryopreserved tumor pieces were cut in 6-µm-thick sections, fixed in ice cold acetone for 5 min, and air-dried. Slides were then blocked in PBS with 3% BSA at RT for 5 min. For staining of EDB, 1 µg of the respective biotinylated cystine-knot miniprotein was incubated with 2.9 µg streptavidin-Cy3 conjugate (Rockland Immunochemicals) at RT for 30 min. The pre-formed complex or 0.1 µg AF680-(MC-FN-010)_3_ and anti-mouse CD31 antibody (RB-10333-P1, Thermo Fisher) diluted 1:100 in PBS with 1% BSA was then added to the tumor sections and incubated for 30 min at 37 °C. Afterwards, slides were washed three times with PBS containing 1% BSA. CD31 staining of sections treated with pre-formed complex was performed with a rat anti-mouse CD31 IgG antibody (102416; BioLegend) diluted 1:100 in PBS with 1% BSA for 30 min at 37 °C and detected with a secondary anti-rabbit IgG-Cy3 antibody (111-165-003; Jackson ImmunoResearch) diluted 1:400 in PBS with 1% BSA for 30 min at 37 °C. After three washing steps in PBS, cell nuclei were stained with Höchst 33342 (Thermo Fisher Scientific) diluted 1:5000 in PBS for 30 min at RT. Slides were washed again as described above and covered with coverslips in a thin layer of mounting medium (Dako). Images were captured with a Zeiss Apotome microscope (Carl Zeiss) and analyzed with ZEN software (Carl Zeiss).

### Peptide synthesis

Trimeric ligands as well as N-terminally biotinylated miniproteins were purchased from Pepscan. Cystine-knot miniproteins were generated via conventional solid phase peptide synthesis (SPPS). The oligomerization strategy of trimeric ligands was similar to the described procedure of Kim et al.^44^, but using a triple GSGSK-S spacer peptide that served as a trimerization handle. AF680-labeled aptide and nonapeptide was synthesized according to the same protocol of Kim et al.^17^ or Han et al.^19^, respectively. All obtained peptidic constructs are listed in Supplementary Table 3 and stored as 100 µg aliquots at −20 °C. For experiments all peptides were dissolved in 100 µL DPBS (Gibco) resulting in a concentration of 1 µg/µL. For all constructs identity was verified by ESI mass spectrometry and purity was analyzed by analytical reverse phase chromatography (performed by Pepscan, see Supplementary Figs. 9 and 10 and Supplementary Tables 3 and 4). Additionally, trimers were analyzed via SDS-PAGE and SPR in order to characterize target-binding properties (binding to FN-67B89, see Supplementary Fig. 3b) and specificity (binding to FN-6789).

### U-87 MG xenograft mouse model

Human glioblastoma U-87 MG (ATCC) cell line was cultured in EMEM medium (ATCC) supplemented with 10% FCS under aseptic conditions at 37 °C with 5% CO_2_ and 95% humidity. Cell lines from ATCC have been thoroughly analyzed and authenticated to ensure their identity and quality. Additionally, we verified the cell line via morphology examination, NGS sequencing, and STR profiling and tested them negatively for mycoplasma contamination. All mice were kept in accordance with federal and state policies on animal research at BioNTech SE. Procedures and experimental group sizes were approved by the regulatory authorities (Tierschutzkommision des Landesuntersuchungsamts Rheinland-Pfalz) for animal welfare. Four-week-old Fox n1/nu mice ranging in weights between approximately 25 and 28 g were obtained from Janvier Labs. For xenograft mouse studies 7 × 10^6^ human U-87 MG cells were subcutaneously injected into the right flank of Fox n1/nu mice and tumors were allowed to grow for approximately 5 weeks. Subcutaneous tumor size was determined using ellipsoid formula ((width × length^2^)/2)). All animals with tumor volume between 50 and 1500 mm^3^ were included in the studies and mice were randomly assigned to experimental cohorts.

### In vivo and ex vivo imaging

Mice carrying a desired tumor size were included for analysis of biodistribution and tumor targeting of trimeric constructs and peptides. All trimeric constructs and peptides were injected intravenously via retrobulbar venous plexus in a final volume of 100 µL PBS buffer (3.34 nmol, 10 nmol, or 34.7 nmol/mice). Mice (*n* = 3 for each construct) were imaged in an IVIS Spectrum System (Perkin Elmer) using excitation range of 615–665 nm and monitoring emission signals at 695–770 nm. Imaging process was performed 1, 2, 3, or 6 h post-injection and after euthanization the tumor and specific organs were excised, imaged, weighed, and cryo-conservated for further analysis. Fluorescence intensity of regions of interest was quantified using Living Image^®^ software (Perkin Elmer).

### Statistical analysis

Statistical significance was calculated based on triplicate data sets using two-way ANOVA analysis with corrected *p* values (Sidak’s method) for comparison of compounds and between tissue types in GraphPad Prism 6. *p < 0.05; **p < 0.01; ***p < 0.001. All data are presented as mean ± SD.

### Reporting summary

Further information on research design is available in the Nature Research Reporting Summary linked to this article.

## Supplementary information

Supplementary Information

Reporting Summary

## Data Availability

The source data underlying Figs. 2, 3, 4 and Supplementary Figs. 1, 2, 3, 4, 7 are provided as a Source Data file. Any other data are available from the authors upon reasonable request.

## Source Data

Source Data
