# Supplementary material for: Targeting the tumor vasculature with engineered cystine-knot miniproteins
Source: Nat Commun. 2020 Jan 15;11:295. doi: 10.1038/s41467-019-13948-y (PMC6962393; doi:10.1038/s41467-019-13948-y)
Supplement: Supplementary file 1 — Supplementary Information [file 41467_2019_13948_MOESM1_ESM.pdf]

Supplementary Information

Targeting the tumor vasculature with engineered cystine-knot miniproteins

Lui et al.

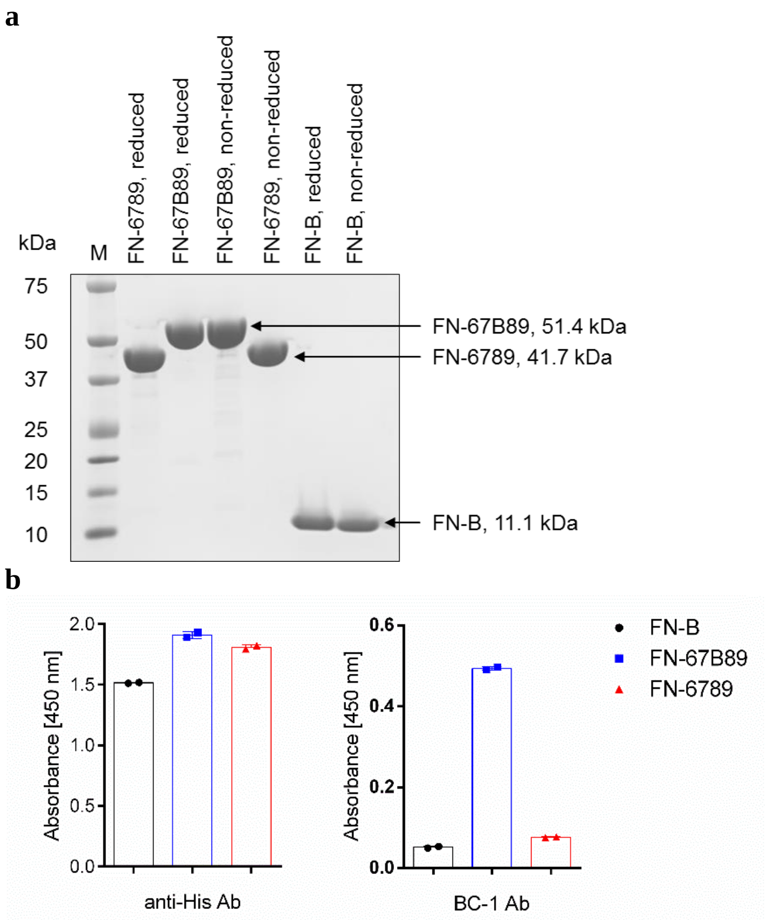

Supplementary Figure 1 Quality control of recombinantly produced FN-proteins. (a) SDS-PAGE of FN-proteins after IMAC and SEC purification. A total of 10 µg protein per lane was applied under reducing (+β-mercaptoethanol) and non-reducing (-β-mercaptoethanol) conditions. **(b)** ELISA-based binding analysis for confirmation of identity (anti-His-tag) and folding (BC-1), respectively. 1 µg protein was coated per well. Error bars represent mean ± SD. Source data are provided as a Source Data file.

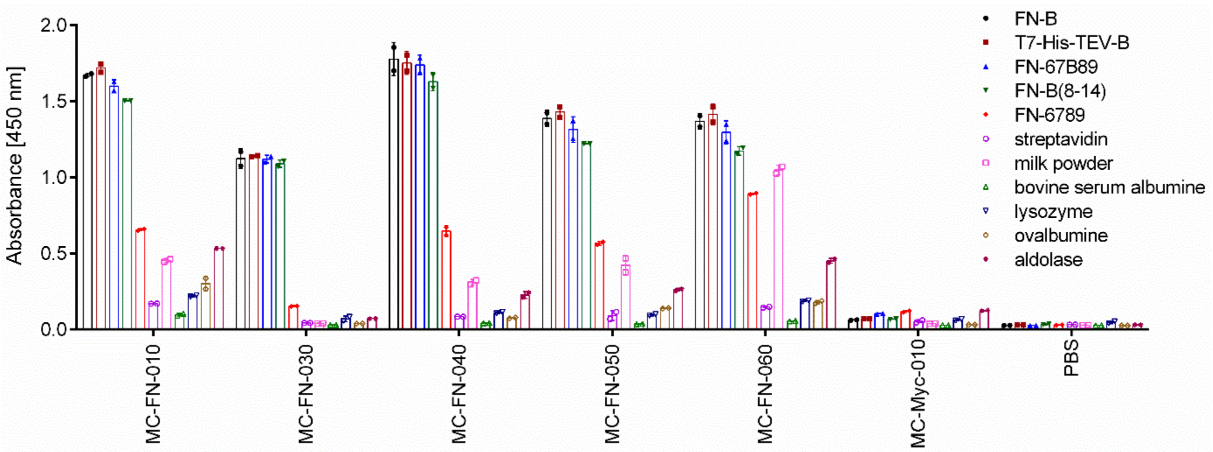

Supplementary Figure 2 Specificity analysis of selected cystine-knot miniproteins. Extended specificity analysis of selected cystine-knot miniproteins. 200 nM of each Trx-cystine-knot miniproteins were applied to immobilized FN-B, T7-His-TEV-B, FN-67B89 and FN-B(8-14) target proteins and the negative controls FN-6789, streptavidin, milk powder, bovine serum albumin, lysozyme, ovalbumin and aldolase (coated at 1 µg/well). Data are shown as mean ± SD. Source data are provided as a Source Data file.

a

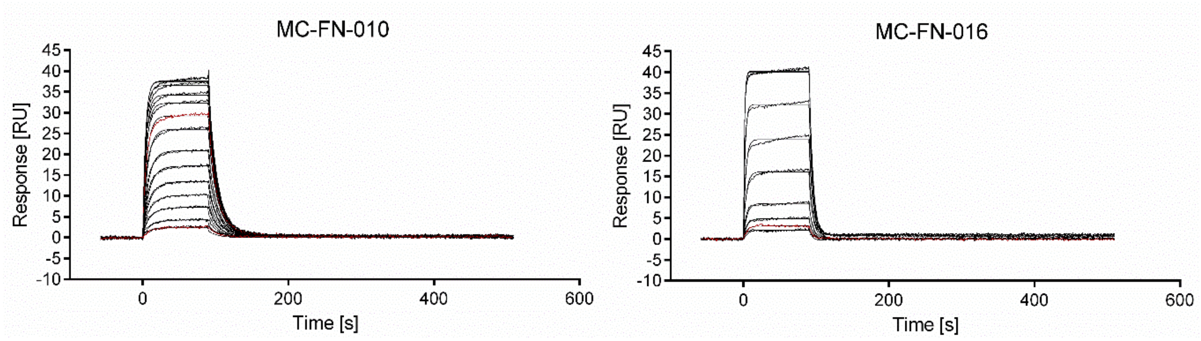

b

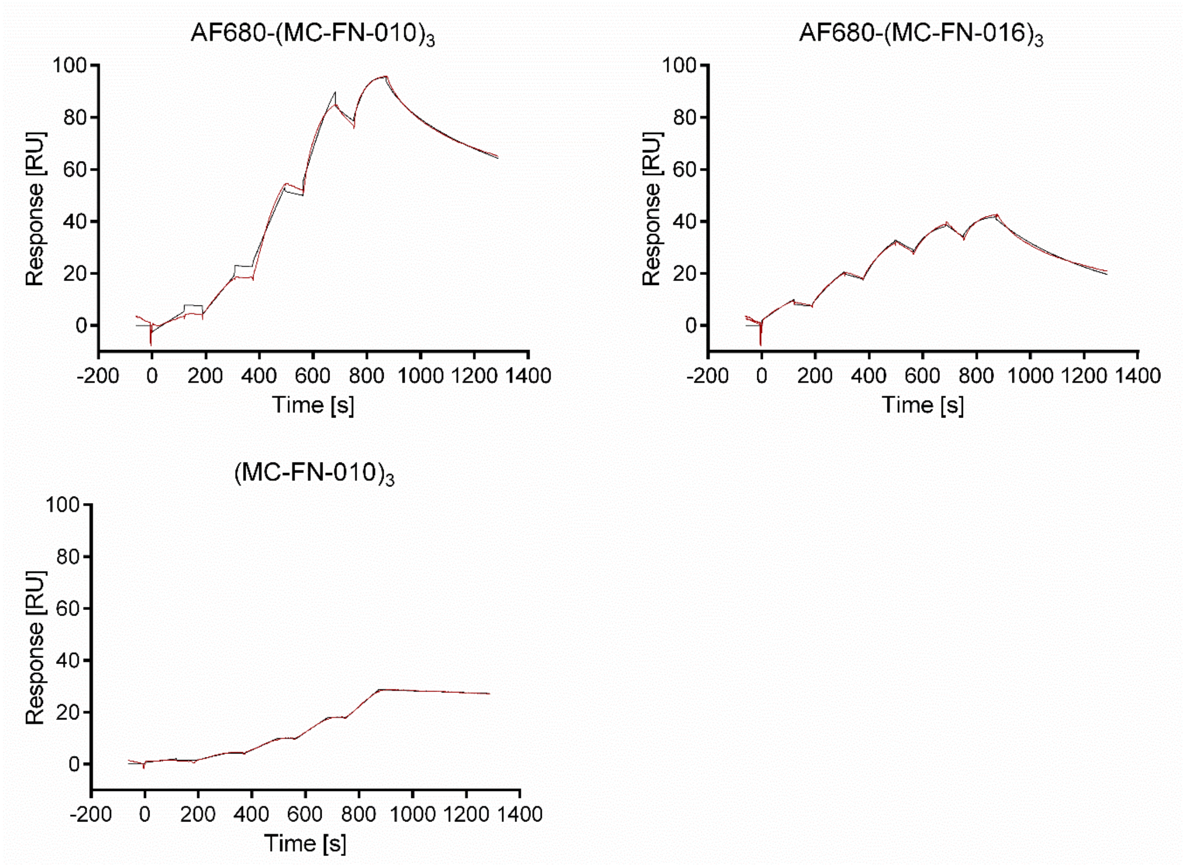

Supplementary Figure 3 SPR sensorgrams of cystine-knot miniprotein variants to EDB. (a) Sensorgrams resulting from multi-cycle kinetic analysis of monomeric MC-FN-010 and MC-FN-016 binding to biotinylated FN-67B89 captured on streptavidin sensor chip. Concentrations ranging from 50 nM to 4000 nM for MC-FN-010 and 62.5 nM to 4000 nM for MC-FN-016 (curves from bottom down to top) were applied. The curves were corrected to a blank streptavidin control flow cell. Kinetic parameters obtained by applying a 1:1 Langmuir fitting model are shown in Table 1. **(b)** Sensorgrams arising from a single-cycle kinetic analysis of AF680-(MC-FN-010)$_3$, AF680-(MC-FN-016)$_3$ and (MC-FN-010)$_3$ binding to biotinylated FN-67B89 captured on streptavidin sensor chip. Different concentrations of trimeric constructs from 1.25 to 10 nM were subsequently analyzed. The sensorgrams were referenced to a blank streptavidin control flow cell. Kinetic parameters obtained by applying a 1:1 Langmuir fitting model are depicted in Table 1. Source data are provided as a Source Data file.

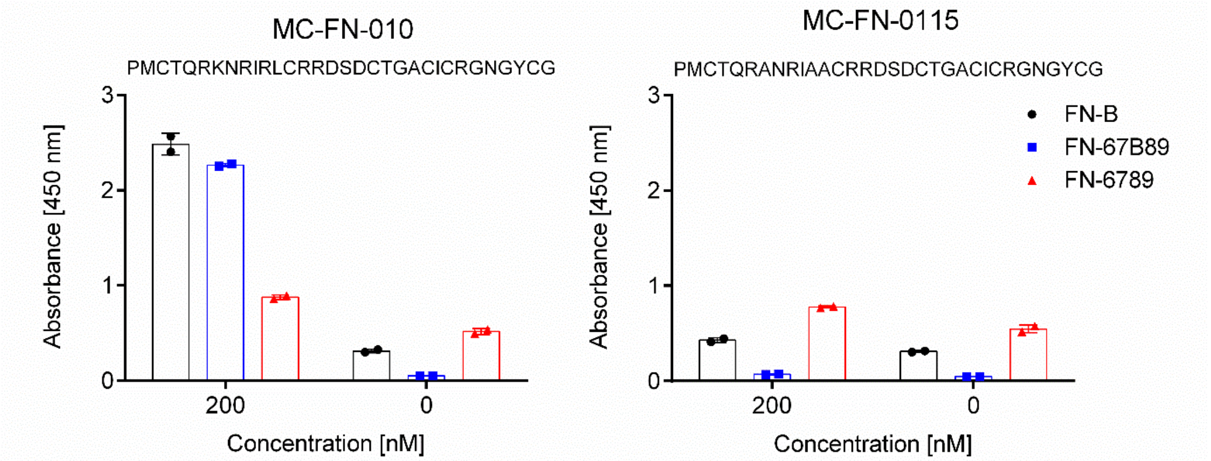

Supplementary Figure 4 Binding analysis of MC-FN-010 and negative control MC-FN-0115. ELISA-based binding analysis of Trx-cystine-knot miniproteins with applied concentration at 200 nM to coated FN-B and FN-67B89 target proteins and to the control protein FN-6789 (coated at 1 µg/well). Data are shown as mean ± SD. Source data are provided as a Source Data file.

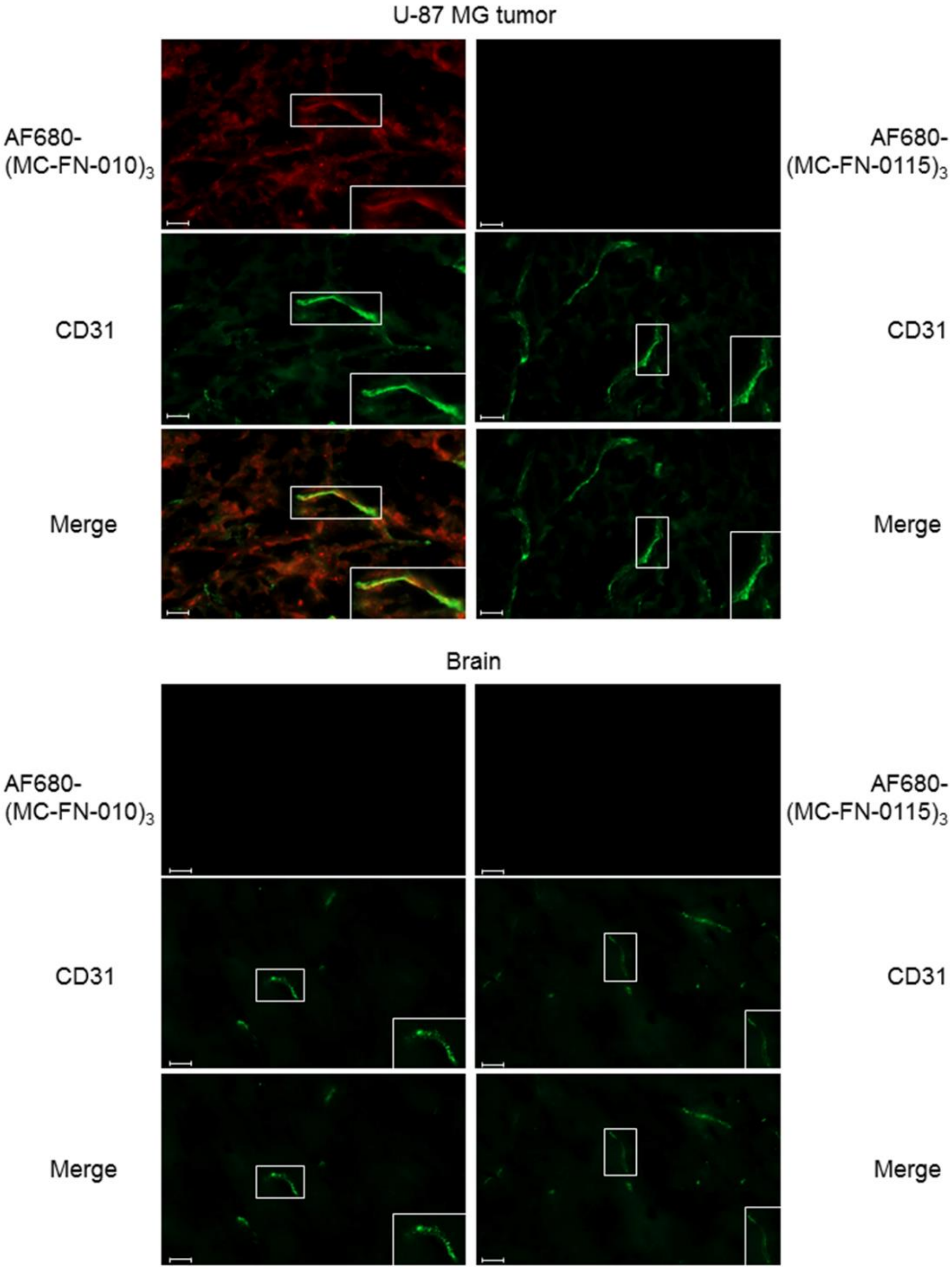

Supplementary Figure 5 Specific binding of trimeric MC-FN-010 to tumor tissue sections. Tissue sections were derived from the human U-87 MG glioblastoma cell line grown as mouse xenograft tumor. Representative immunofluorescence staining of U-87 MG tumor tissues and normal mouse brain with trimeric MC-FN-010 and negative control MC-FN-0115. Tissue sections (6 µm) were stained with Alexa Fluor 680 conjugated trimeric cystine-knot miniproteins (red) and an anti-CD31 antibody detected with a secondary antibody to visualize vasculature (green). Scale bars, 20 µm.

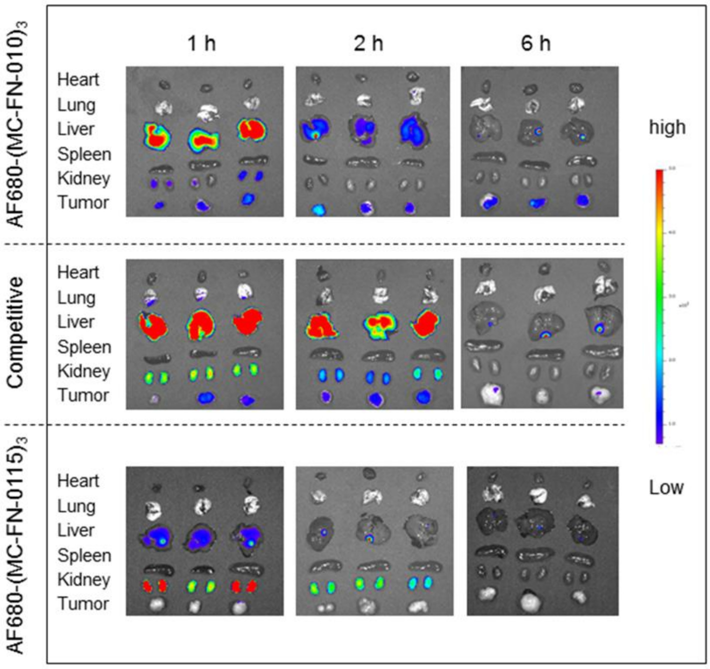

Supplementary Figure 6 Specific tumor targeting with cystine-knot miniprotein. Ex vivo images of organs and U-87 MG xenograft tumors. Images were taken after i.v. application of 3.34 nmol AF680-$(MC\text{-}FN\text{-}010)_3$ alone or administered 30 min after pre-injection of a 10-fold molar excess of $(MC\text{-}FN\text{-}010)_3$ (Competitive) and negative control AF680-$(MC\text{-}FN\text{-}0115)_3$.

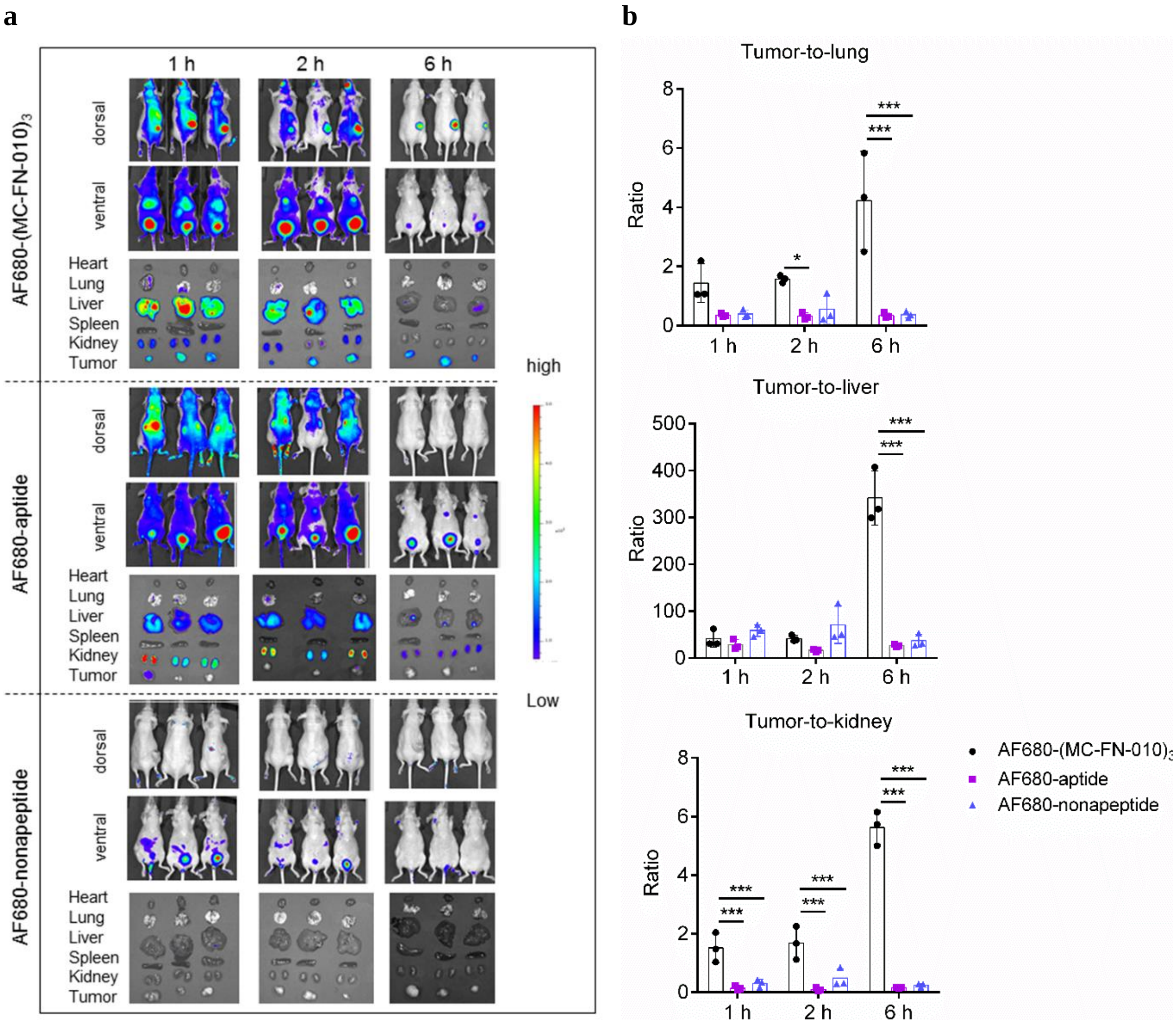

Supplementary Figure 7 Superior in vivo imaging performance of cystine-knot miniproteins. (a) In vivo and ex vivo images of mice with tumors arising from U-87 MG cells and i.v. injected AF680-(MC-FN-010)$_3$, AF680-aptide and AF680-nonapeptide (3.34 nmol). Groups of three mice were stratified to represent a range of tumor sizes. **(b)** Kinetics of tumor-to-lung, tumor-to-liver and tumor-to-kidney ratio. Ratios of ex vivo images were calculated from normalized fluorescence signals after i.v. injection of 3.34 nmol AF680-(MC-FN-010)$_3$, AF680-aptide and AF680-nonapeptide at 1 h, 2 h and 6 h (n=3). Data are shown as mean ± SD of triplicate and statistical analysis was performed using two-way ANOVA (* p< 0.05; ** p<0.01; *** p<0.001). Source data are provided as a Source Data file.

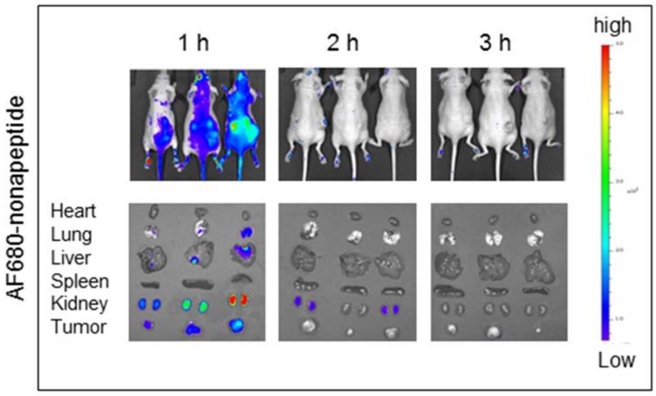

Supplementary Figure 8 In vivo binding of nonapeptide in U87-MG mice xenografts. In vivo and ex vivo images of U-87 MG bearing mice (n=3/group) were measured after i.v. injection of 10 nmol AF680-nonapeptide.

**Mass spectrum**	**UV chromatogram**
AF680-(MC-FN-010)$_3$	AF680-(MC-FN-010)$_3$

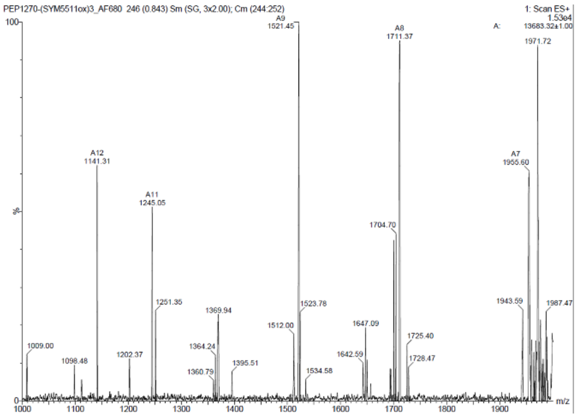

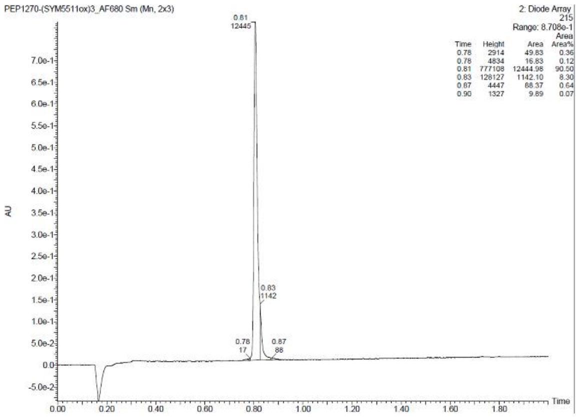

AF680-(MC-FN-016)$_3$	AF680-(MC-FN-016)$_3$

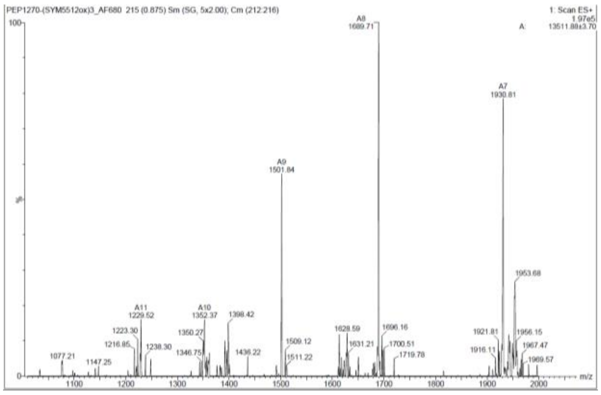

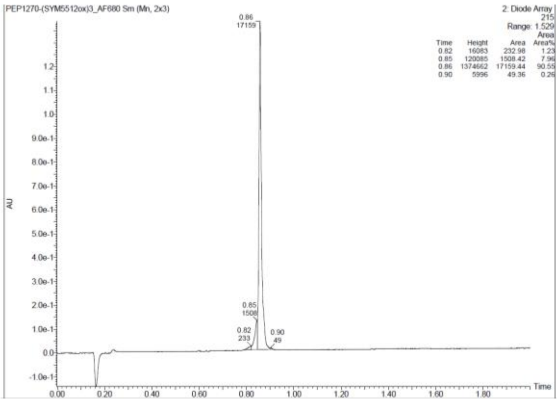

(MC-FN-010)$_3$	(MC-FN-010)$_3$

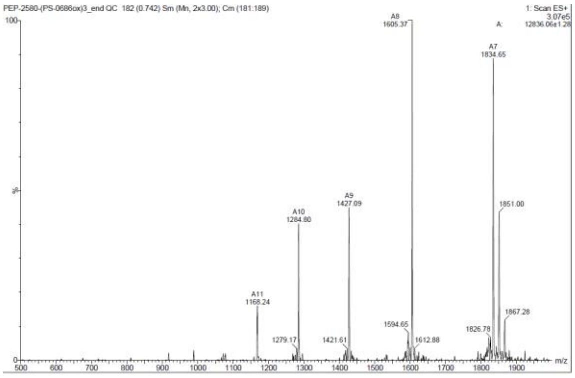

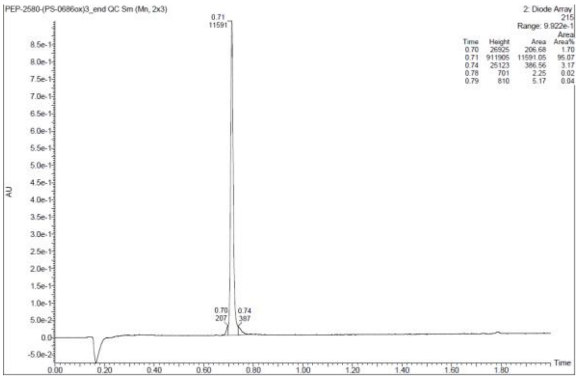

AF680-(MC-FN-0115)$_3$	AF680-(MC-FN-0115)$_3$

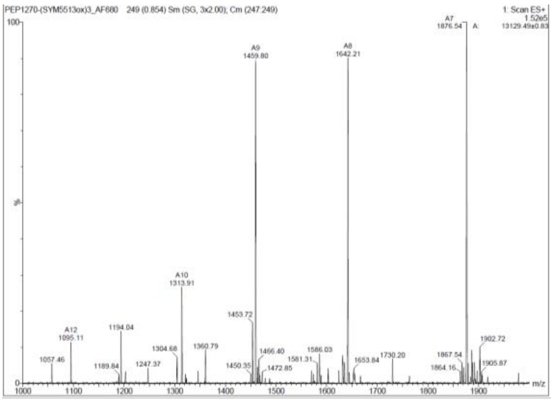

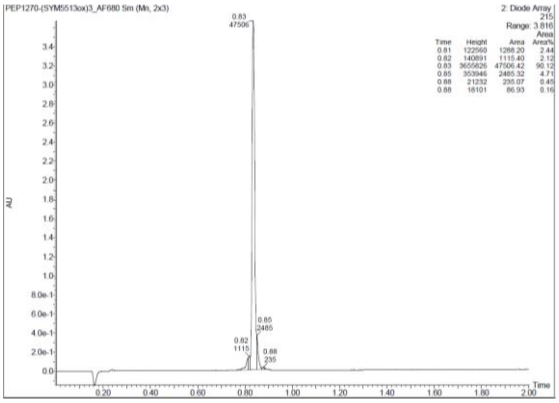

Supplementary Figure 9 Mass spectrometry analysis of cystine-knot miniprotein variants. Mass spectrum and UV chromatogram of trimeric cystine-knot miniprotein variants.

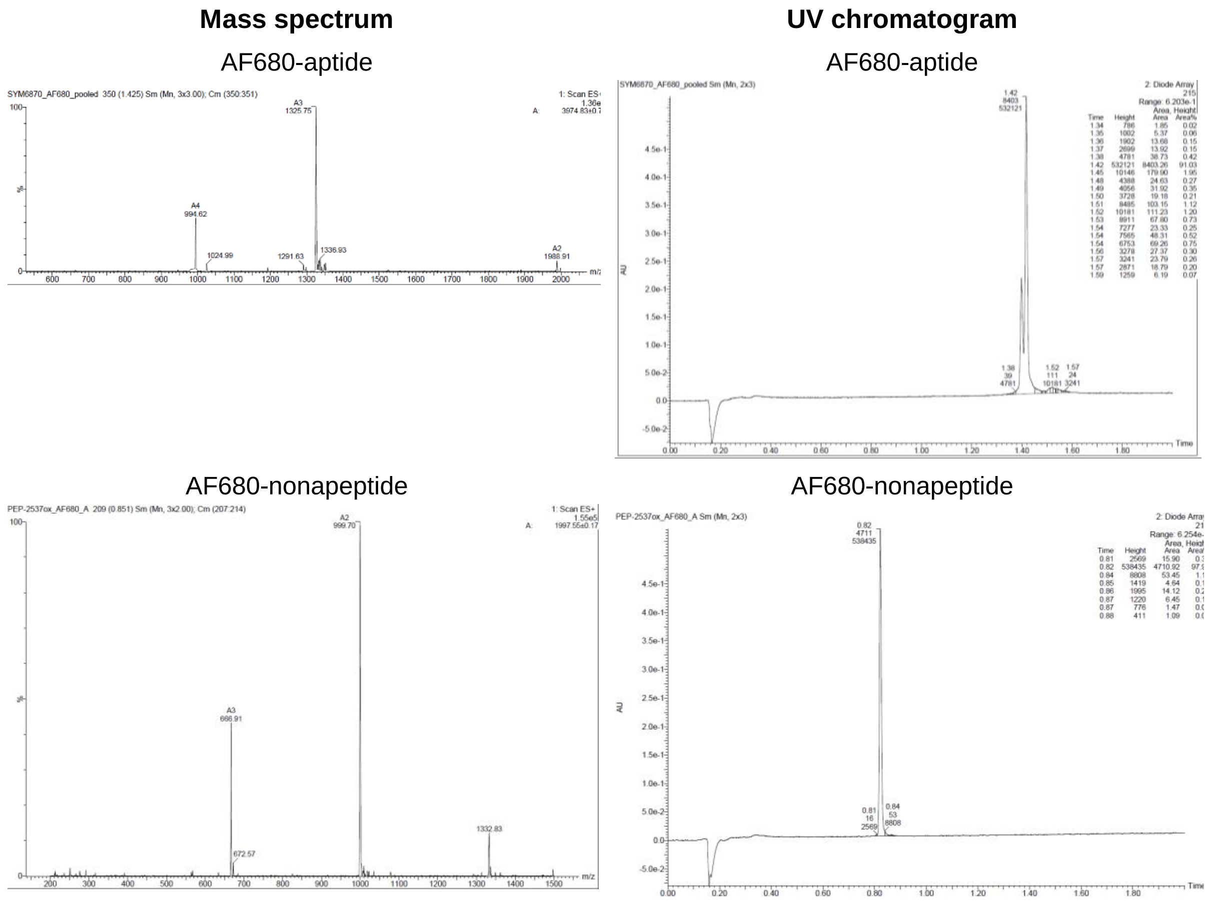

Supplementary Figure 10 Mass spectrometry analysis of aptide and nonapeptide. Mass spectrum and UV chromatogram of aptide and nonapeptide.

Clone name	Ranking value	Protein sequence
MC-FN-020	3.7 to 1.7	WTCTKKYPNTISCRRDSDCRVTCICRGNGYCG
MC-FN-030	2.7 to 1.6	WKCQPTNGYRIRCRRDSDCPGDCICRGNGYCG
MC-FN-010	2.6 to 0.6	PMCTQRKNRIRLCRRDSDCTGACICRGNGYCG

Supplementary Table 1 Selected EDB-binding cystine-knot miniproteins from MCopt 1.0 library. EDB-binding cystine-knot miniproteins were enriched via phage display screening and traverse the hit identification process. Ranking scores were calculated on the basis of the signal (FN-B) to noise (BSA) ratios determined via ELISA and were further normalized to the expression rate of Trx-cystine-knot miniproteins in order to compensate concentration differences.

Peptidic construct	Tumor-to-organ ratio	1 h	2 h	6 h
AF680-(MC-FN-010)$_3$	Tumor-to-heart	10.57 ± 4.23	9.31 ± 1.87	15.20 ± 2.98
AF680-(MC-FN-010)$_3$	Tumor-to-lung	1.44 ± 0.65	1.57 ± 0.12	4.22 ± 1.67
AF680-(MC-FN-010)$_3$	Tumor-to-liver	41.19 ± 18.32	41.57 ± 6.70	341.55 ± 57.37
AF680-(MC-FN-010)$_3$	Tumor-to-spleen	4.36 ± 1.07	3.45 ± 0.96	7.15 ± 1.67
AF680-(MC-FN-010)$_3$	Tumor-to-kidney	1.52 ± 0.50	1.68 ± 0.57	5.62 ± 0.58
AF680-aptide	Tumor-to-heart	2.14 ± 0.17	1.73 ± 0.33	1.59 ± 0.34
AF680-aptide	Tumor-to-lung	0.35 ± 0.05	0.31 ± 0.12	0.34 ± 0.09
AF680-aptide	Tumor-to-liver	28.55 ± 10.79	16.68 ± 2.46	26.80 ± 3.13
AF680-aptide	Tumor-to-spleen	0.83 ± 0.05	0.77 ± 0.16	0.46 ± 0.10
AF680-aptide	Tumor-to-kidney	0.14 ± 0.08	0.10 ± 0.05	0.16 ± 0.01
AF680-nonapeptide	Tumor-to-heart	2.12 ± 1.18	2.21 ± 1.62	0.97 ± 0.15
AF680-nonapeptide	Tumor-to-lung	0.40 ± 0.14	0.55 ± 0.48	0.37 ± 0.11
AF680-nonapeptide	Tumor-to-liver	58.82 ± 12.65	71.14 ± 39.80	37.03 ± 13.96
AF680-nonapeptide	Tumor-to-spleen	0.74 ± 0.35	0.84 ± 0.53	0.43 ± 0.22
AF680-nonapeptide	Tumor-to-kidney	0.31 ± 0.13	0.49 ± 0.32	0.25 ± 0.06

Supplementary Table 2 Biodistribution of peptidic constructs in human U-87 MG xenograft mouse tumor. Tumor-to-organ ratios of AF680-(MC-FN-010)$_3$, AF680-aptide and AF680-nonapeptide were calculated based on the fluorescence signals (normalized to the weight) after i.v. injection of 3.34 nmol samples at 1 h, 2 h and 6 h (n=3). Data are shown as mean ± SD of triplicates.

Peptidic construct	Molecular weight (Da)	Purity (%)
AF680-(MC-FN-010)$_3$	13681.9	90.5
AF680-(MC-FN-016)$_3$	13510.6	90.5
(MC-FN-010)$_3$	12839.2	95.0
AF680-(MC-FN-0115)$_3$	13129.0	90.1
MC-FN-010-bio	4171.9	95.5
MC-FN-0115-bio	4168.7	94.9
AF680-aptide	3977.2	91.0
AF680-nonapeptide	2000.3	97.9

Supplementary Table 3 Molecular weight and purity of peptidic constructs. Purities of trimeric cystine-knot miniprotein variants, aptide and nonapeptide were calculated based on analytical reversed-phase chromatography. The corresponding UV-vis chromatograms are shown in supplementary Figure 9 and Figure 10.

Peptidic construct	Molecular mass (Da)	Expected mass	Detected mass
AF680-(MC-FN-010)$_3$	13681.9	2281.32 (2 H^+)	
AF680-(MC-FN-010)$_3$	13681.9	1521.22 (3 H^+)	1521.45 (3 H^+)
AF680-(MC-FN-010)$_3$	13681.9	1141.17 (4 H^+)	
AF680-(MC-FN-016)$_3$	13510.6	2252.77 (2 H^+)	
AF680-(MC-FN-016)$_3$	13510.6	1502.19 (3 H^+)	1501.84 (3 H^+)
AF680-(MC-FN-016)$_3$	13510.6	1459.79 (4 H^+)	
AF680-(MC-FN-0115)$_3$	13129.0	2189.17 (2 H^+)	
AF680-(MC-FN-0115)$_3$	13129.0	1459.79 (3 H^+)	1459.80 (3 H^+)
AF680-(MC-FN-0115)$_3$	13129.0	1095.09 (4 H^+)	
(MC-FN-010)$_3$	12839.2	2140.87 (2 H^+)	
(MC-FN-010)$_3$	12839.2	1427.59 (3 H^+)	1427.09 (3 H^+)
(MC-FN-010)$_3$	12839.2	1070.94 (4 H^+)	
AF680-aptide	3977.2	1989.61 (2 H^+)	1988.01 (2 H^+)
AF680-aptide	3977.2	1326.74 (3 H^+)	1325.75 (3 H^+)
AF680-aptide	3977.2	995.31 (4 H^+)	994.62 (4 H^+)
AF680-nonapeptide	2000.3	1001.16 (2 H^+)	999.7 (2 H^+)
AF680-nonapeptide	2000.3	667.77 (3 H^+)	666.91 (3 H^+)
AF680-nonapeptide	2000.3	666.91 (4 H^+)	

Supplementary Table 4 Mass spectrometry analysis of peptidic constructs Expected mass of two-, three- and four-fold charged trimeric cystine-knot miniproteins variants, aptide and nonapeptide (2 H^+, 3 H^+, 4 H^+) and the actual detected mass resulting from mass spectrometry. The corresponding mass spectra are shown in supplementary Figure 9 and Figure 10.
