# Supplementary material for: Targeting the tumor vasculature with engineered cystine-knot miniproteins
Source: Nat Commun. 2020 Jan 15;11:295. doi: 10.1038/s41467-019-13948-y (PMC6962393; doi:10.1038/s41467-019-13948-y)
Supplement: Supplementary file 2 — Reporting Summary [file 41467_2019_13948_MOESM2_ESM.pdf]

nature research

Corresponding author(s): Ugur Sahin

Last updated by author(s): Nov 7, 2019

Reporting Summary

Nature Research wishes to improve the reproducibility of the work that we publish. This form provides structure for consistency and transparency in reporting. For further information on Nature Research policies, see Authors & Referees and the Editorial Policy Checklist .

Statistics

For all statistical analyses, confirm that the following items are present in the figure legend, table legend, main text, or Methods section.

n/a	Confirmed	
☐	☒	The exact sample size (*n*) for each experimental group/condition, given as a discrete number and unit of measurement
☐	☒	A statement on whether measurements were taken from distinct samples or whether the same sample was measured repeatedly
☐	☒	The statistical test(s) used AND whether they are one- or two-sided<br>*Only common tests should be described solely by name; describe more complex techniques in the Methods section.*
☐	☒	A description of all covariates tested
☐	☒	A description of any assumptions or corrections, such as tests of normality and adjustment for multiple comparisons
☐	☒	A full description of the statistical parameters including central tendency (e.g. means) or other basic estimates (e.g. regression coefficient) AND variation (e.g. standard deviation) or associated estimates of uncertainty (e.g. confidence intervals)
☐	☒	For null hypothesis testing, the test statistic (e.g. *F*, *t*, *r*) with confidence intervals, effect sizes, degrees of freedom and *P* value noted<br>*Give P values as exact values whenever suitable.*
☒	☐	For Bayesian analysis, information on the choice of priors and Markov chain Monte Carlo settings
☒	☐	For hierarchical and complex designs, identification of the appropriate level for tests and full reporting of outcomes
☒	☐	Estimates of effect sizes (e.g. Cohen's *d*, Pearson's *r*), indicating how they were calculated

Our web collection on statistics for biologists contains articles on many of the points above.

Software and code

Policy information about availability of computer code

Data collection	n/a
Data analysis	n/a

For manuscripts utilizing custom algorithms or software that are central to the research but not yet described in published literature, software must be made available to editors/reviewers. We strongly encourage code deposition in a community repository (e.g. GitHub). See the Nature Research guidelines for submitting code & software for further information.

Data

Policy information about availability of data

All manuscripts must include a data availability statement. This statement should provide the following information, where applicable:

- Accession codes, unique identifiers, or web links for publicly available datasets
- A list of figures that have associated raw data
- A description of any restrictions on data availability

Source data are provided as a Source Data file.

Field-specific reporting

Please select the one below that is the best fit for your research. If you are not sure, read the appropriate sections before making your selection.

☒ Life sciences ☐ Behavioural & social sciences ☐ Ecological, evolutionary & environmental sciences

For a reference copy of the document with all sections, see nature.com/documents/nr-reporting-summary-flat.pdf

Life sciences study design

All studies must disclose on these points even when the disclosure is negative.

Sample size	For the in vivo study a cohort of 3 mice was determined in order to cover a statistically significant number.<br>For in vitro experiments the sample size was at least n=2.
Data exclusions	n/a
Replication	figure 2b: n=2, no experimental repetition, in the course of first hit-screening only clones with target-interaction were chosen for second hit-ELISA<br>figure 2c: n=2, experimental repetition with variation of applied concentrations<br>figure 2d: MC-FN-010 --> experimental repetition, MC-FN-030, MC-FN-040 and MC-FN-050: these constructs were excluded for further analysis due to no detectable binding of even high concentration (1 µM)<br>figure 3a: n=2, experimental repetition<br>figure 3b: n=2, experimental repetition<br>figure 4c: n=3, no experimental repetition with n=3, but similar experiment with smaller cohort<br><br>figure S1b: n=2, experimental repetition<br>figure S2: n=2, no experimental repetition, in the course of downstream hit-identification we performed a second hit-ELISA with the clones resulting from the first hit-ELISA. Subsequently, the most promising candidates were further characterized (e. g. SPR analysis)<br>figure S3: n=2, experimental repetition<br>figure S4: experimental repetition
Randomization	figure 4b: Mice were stratified and randomly assigned to groups<br>figure 4e, figure S8: Mice were stratified and randomly assigned to groups<br>figure S9: Mice were stratified and randomly assigned to groups<br>figure S10: Mice were stratified and randomly assigned to groups
Blinding	n/a

Reporting for specific materials, systems and methods

We require information from authors about some types of materials, experimental systems and methods used in many studies. Here, indicate whether each material, system or method listed is relevant to your study. If you are not sure if a list item applies to your research, read the appropriate section before selecting a response.

Materials & experimental systems

n/a	Involved in the study	
☐	☒	Antibodies
☐	☒	Eukaryotic cell lines
☒	☐	Palaeontology
☐	☒	Animals and other organisms
☒	☐	Human research participants
☒	☐	Clinical data

Methods

n/a	Involved in the study	
☒	☐	ChIP-seq
☒	☐	Flow cytometry
☒	☐	MRI-based neuroimaging

Antibodies

Antibodies used	1. Anti-Fibronectin antibody [BC-1], supplier name: abcam, cat-no: ab154210, clone name: BC-1, Lot-no: GR296651-2<br>2. Anti-S tag antibody (HRP), supplier name: Abcam, cat-no: ab19324<br>3. Anti-S tag antibody (HRP), supplier name: Abcam, cat-no: ab18589<br>4. Alexa Fluor® 647 anti-mouse CD31 Antibody, supplier name: BioLegend®, cat-no: 102416 , clone name: clone 390, Lot-no: B239267<br>5. Anti-rabbit IgG-Cy3 antibody, supplier name: Jackson ImmunoResearch, cat-no: 111-165-003<br>6. Anti-mouse CD31 antibody, supplier name: Thermo Fisher, cat-no: RB-10333-P1, Lot-no: 10333P111OU<br>7. Anti-His tag antibody (HRP), supplier name: Dianova, cat-no: DIA-900, Lot-no: 13618B
Validation	1. suitable for ELISA and reacts with human Fibronectin+EDB: https://www.abcam.com/fibronectin-antibody-bc-1-ab154210.html<br>2. suitable for ELISA to detect synthetic S-tag: https://www.abcam.com/s-tag-antibody-hrp-ab19324.html<br>3. suitable for ELISA to detect synthetic S-tag: https://www.citeab.com/antibodies/707288-ab18589-anti-s-tag-antibody-hrp<br>4. validated for IF to react with mouse CD31: https://www.biolegend.com/en-us/products/alexa-fluor-647-anti-mouse-cd31-antibody-3092

5. Applied as secondary antibody for immunostaining: https://journals.plos.org/plosone/article?id=10.1371/journal.pone.0057038
6. suitable for IHC staining: https://www.thermofisher.com/document-connect/document-connect.html?url=https%3A%2F%2Fassets.thermofisher.com%2FTFS-Assets%2FAPD%2FSpecification-Sheets%2FD12613~.pdf&title=Q0QzMS9QRUNBTS0xIChFbmRvdGhlbGlhbCBDZWxsIE1hcmtlciksIFJhYmJpdCBQb2x5Y2xvbmFsIEFudGlib2R5
7. suitable for ELISA: https://www.dianova.com/produkte/dia-900-200-anti-his-epitop-tag-rek-aus-maus-klon-13-45-31-unkonj-200-%C2%B5g/ (see data sheet)

Eukaryotic cell lines

Policy information about cell lines

Cell line source(s)	U-87 MG (purchased from ATCC®)
Authentication	Cell lines from ATCC have been thoroughly analyzed and authenticated to ensure their identity. Additionally, we verified the cell line via morphology examination, NGS sequencing and STR profiling.
Mycoplasma contamination	We confirm that the cell line was negative for mycoplasma contamination.
Commonly misidentified lines (See ICLAC register)	U-87 MG is registered in ICLAC. In order to ensure the identity and quality of the cells, we purchased a fresh vial from ATCC and verified the cell line as described above.

Animals and other organisms

Policy information about studies involving animals; ARRIVE guidelines recommended for reporting animal research

Laboratory animals	Fox n1/nu, female, four weeks old
Wild animals	n/a
Field-collected samples	n/a
Ethics oversight	Tierschutzkommision des Landesuntersuchungsamts Rheinland-Pfalz

Note that full information on the approval of the study protocol must also be provided in the manuscript.
